# Superabsorption of light via quantum engineering

**DOI:** 10.1038/ncomms5705

**Published:** 2014-08-22

**Authors:** K. D. B. Higgins, S. C. Benjamin, T. M. Stace, G. J. Milburn, B. W. Lovett, E. M. Gauger

**Affiliations:** 1 Department of Materials, Oxford University, Oxford OX1 3PH, UK; 2 Centre for Quantum Technologies, National University of Singapore, 3 Science Drive 2, Singapore 117543, Singapore; 3 Centre for Engineered Quantum Systems, School of Mathematics and Physics, The University of Queensland, St Lucia, Queensland 4072, Australia; 4 SUPA, School of Physics and Astronomy, University of St Andrews, St Andrews, KY16 9SS, UK

## Abstract

Almost 60 years ago Dicke introduced the term superradiance to describe a signature quantum effect: *N* atoms can collectively emit light at a rate proportional to *N*^2^. Structures that superradiate must also have enhanced absorption, but the former always dominates in natural systems. Here we show that this restriction can be overcome by combining several well-established quantum control techniques. Our analytical and numerical calculations show that superabsorption can then be achieved and sustained in certain simple nanostructures, by trapping the system in a highly excited state through transition rate engineering. This opens the prospect of a new class of quantum nanotechnology with potential applications including photon detection and light-based power transmission. An array of quantum dots or a molecular ring structure could provide a suitable platform for an experimental demonstration.

## Abstract (web summary)

A quantum system that super-radiates must also exhibit enhanced absorption, but the former always dominates in natural systems. However, by invoking environmental quantum control techniques, Higgins *et al.* demonstrate that a system can exhibit quantum-enhanced light absorption.

## Graphical abstract

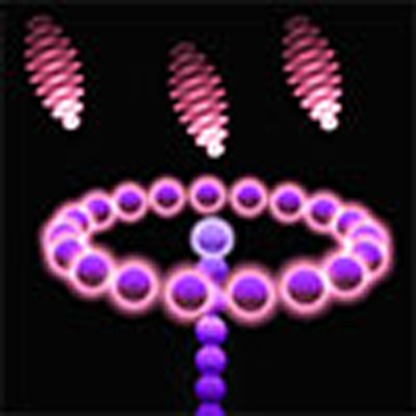

Superradiance can occur when *N* individual atoms interact with the surrounding electromagnetic field^1^. Here we use the term ‘atom’ broadly to refer to entities with a discrete dipole-allowed transition, including semiconductor quantum dots^2^, crystal defects and molecules^3^. Following an initial excitation of all atoms, dipole-allowed decay down a series of symmetrical ‘Dicke ladder’ states leads to an enhanced light–matter coupling that, when the system reaches the state half way down the ladder, depends on the square of the atomic transition dipole^1^,^4^,^5^. Thus when *N* dipoles add coherently, light can be emitted at an enhanced rate proportional to *N*^2^. Even for moderate *N* this represents a significant increase over the prediction of classical physics, and the effect has found applications ranging from probing exciton delocalization in biological systems^6^, to developing a new class of laser^7^ and may even lead to observable effects in astrophysics^8^.

Time-reversal symmetry of quantum mechanics implies that systems with enhanced emission rates will also have enhanced absorption rates. Naturally emission dominates if an excited state of the collective emits into a vacuum, since there are no photons to absorb. Even in an intense light field where absorption and emission are closely balanced, a given transition remains more likely to emit than to absorb. Thus it might seem that the inverse of superradiance is intrinsically ephemeral.

However, here we show that certain interactions between the atoms allow us to control a quantum system such that a sustained superabsorbing state can exist. For atoms in close proximity and with a suitable geometrical arrangement, ever present atomic dipolar interactions are sufficient for our purposes. An appropriate realization involves a ring structure that is strikingly reminiscent of the photosynthetic light harvesting complex LH1 (refs 9, 10; see Fig. 1). Although the potential for enhanced absorption inherently exists in all superradiating systems, natural systems are not designed to ulitize it. Rather, these will always perform an (often strongly) biased random walk down the ladder of accessible states, being attracted by the bottom most rung. Strongly enhanced absorption near the middle of the Dicke ladder is thus an improbable process and can only last for a vanishingly short time.

By contrast, in this Communication, we will show how to harness environmental quantum control techniques to break the dominance of emission over absorption and extend the time during which a collective system maintains the capability for quantum-enhanced absorption. By interfacing the well-established physical phenomena of superradiance, light filtering, photonic band gaps and quantum feedback control, we show that sustained superlinear scaling of the light absorption rate with the number of atoms is possible. Since this represents the reciprocal process to superradiance, we shall refer to it as ‘superabsorption’. Note that this effect is quite distinct from other recent studies investigating collective light–matter interactions in the context of ‘cloaking’^11^ and time-reversed lasing^12^. In the following we present the Dicke model of superradiance before describing the requirements for unlocking engineered superabsorption. Our discussion explores its potential for practical technologies through the examples of photon sensing and light-based energy transmission.

## Results

### Superradiance

The Hamiltonian of an ensemble of *N* identical atoms is (*ħ*=1):


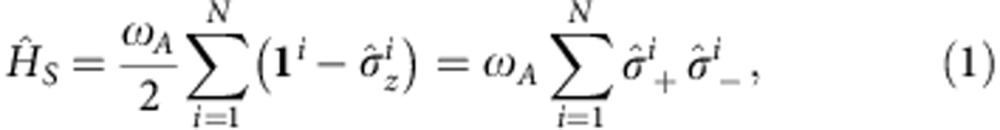


where *ω*_A_ is the bare atomic transition frequency; 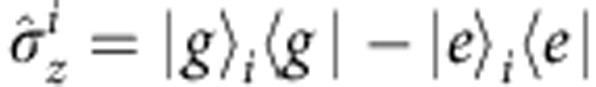, 
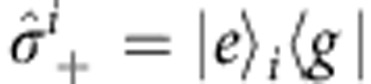
, and 
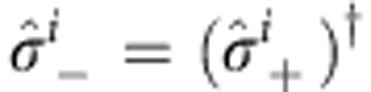
 are the usual Pauli operators defined with respect to the *i*th atom’s ground, |*g*›_*i*_, and optically excited state, |*e*›_*i*_. When the wavelength *λ* of light is much larger than all interatomic distances *r*_*ij*_, (*λ*>>*r*_*ij*_), the atoms become indistinguishable and light interacts with the system collectively. The dynamics are then best described by collective operators:


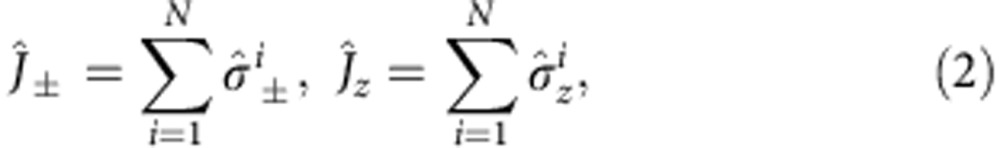


which generate transitions between the eigenstates of the Hamiltonian (1) and obey SU(2) commutation relations. We can succinctly express the light–matter interaction Hamiltonian as


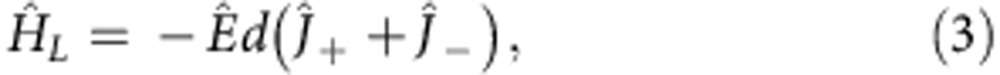


where *Ê* is the light field operator and *d* is the atomic dipole matrix element. The Hamiltonian (3) causes the system to move along a ladder of states called the ‘Dicke’ or ‘bright’ states which are characterized by the eigenvalues *J* and *M* of *Ĵ*^2^ and *Ĵ*_z_, respectively. In the absence of interactions between the atoms, *Ĵ*^2^ commutes with *Ĥ*_S_+*Ĥ*_L_ and thus its eigenvalue 
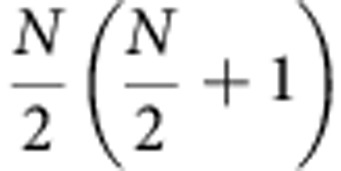
 is a conserved quantity. The Dicke states form a ladder from 
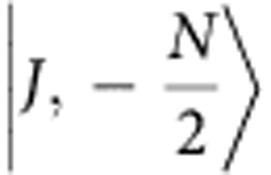
 to 
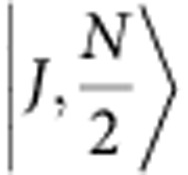
 shown in Fig. 2a; the *N*+1 rungs correspond to the fully symmetric superpositions of *N*/2+*M* excited atoms for each value of *M*. The collective excitation operators


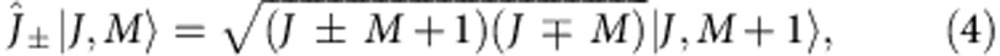


explore this ladder of states, and the transition rates between adjacent Dicke ladder states are then readily calculated:


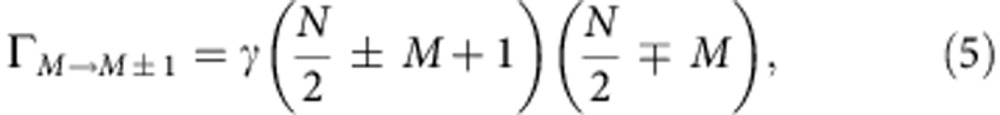


where *γ*=8*π*^2^*d*^2^/(3ε_0_*ħλ*^3^) is the free atom decay rate.

If the system is initialized in the fully excited state 
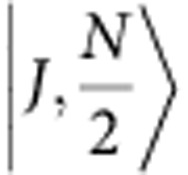
 with no environmental photons, then the system cascades down the ladder, as shown by the red arrows in Fig. 2a. Upon reaching the midway point (*M*=0) its emission rate exceeds the rate *γN* expected of *N* uncorrelated atoms for *N*>2. For a larger number of atoms the peak transition rate of equation (5) follows a quadratic dependence on *N* and is well-approximated by


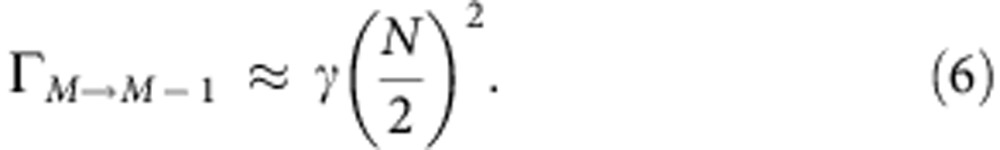


This is the essence of superradiance: constructive interference between the different possible decay paths greatly enhances the emission rate, producing a high intensity pulse. The enhancement is the result of simple combinatorics: near the middle of the ladder, |*J*, 0›, there are a large number of possible configurations of excited atoms that contribute to each respective Dicke state.

Superradiance is not an intrinsically transient effect: steady-state operation can occur through repumping^13^, or in cavities^14^,^15^, and recently a superradiant laser with potential for extraordinary stability and narrow linewidth has been demonstrated^7^.

### Superabsorption

The crucial ingredient for achieving superabsorption is to engineer the transition rates in a way that primarily confines the dynamics to an effective two-level system (E2LS) around the *M*=0 transition (see Fig. 2b), which exhibits the required quadratic absorption rate as depicted in Fig. 3c.

To ensure that most transitions take place within the E2LS we must either suppress the total loss rate from the E2LS or enhance the probability of transitions within it. This becomes possible if the frequency of the E2LS transition is distinct from that of other transitions, and in particular the one immediately below the targeted transition within the E2LS. This will never be the case for a non-interacting set of atoms, which must have a degenerate set of ladder transition energies, but it can occur once suitable interactions are included. Dicke physics requires that the atoms remain identical, but interactions are still permissible in certain symmetric geometries such as rings^4^,^16^, and these structures will continue to exhibit superradiance, and are therefore also capable of superabsorption.

**Figure 1:**
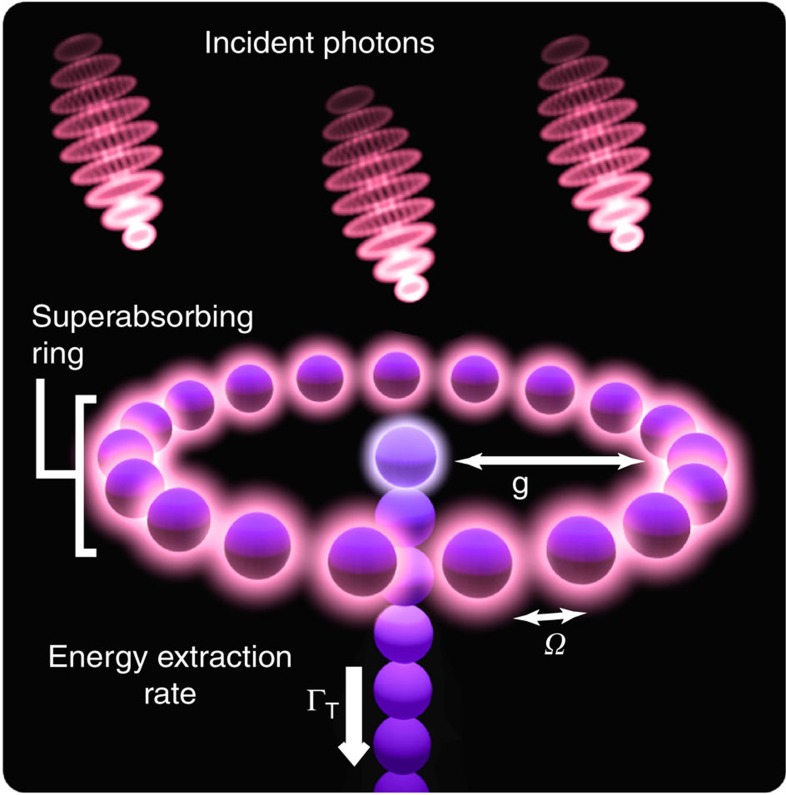
One potential realization of superabsorption. Photons absorbed by the ring give rise to delocalized excitons; ideally the ring maintains a specific exciton population to achieve enhanced absorption. Combined with a suitable charge sensor (for example, a quantum point contact) this enables photon sensing. We also model an application for photon harvesting, where newly created excitons are transferred from the ring to a central core absorber, followed by an irreversible process (for example, one-way transfer down a strongly coupled chain) to a centre converting the exciton into stored energy.

To show this, we consider the candidate superabsorber depicted in Fig. 1. We assume that the interactions act between adjacent atoms only and are due to Förster-type coupling. This leads to a Dicke ladder of non-degenerate transitions whose dynamics are found from a collective quantum optical master equation:


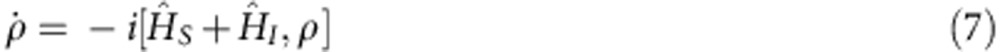



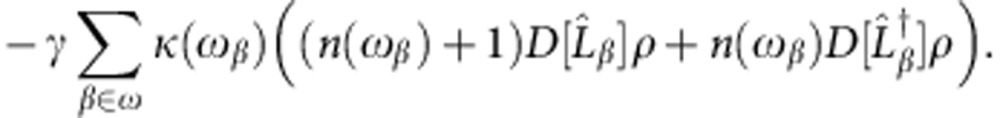


*κ*(*ω*)=∑_*k*_|*g*_*k*_|^2^*δ*(*ω*−*ω*_*k*_)≡*χ*(*ω*)|*g*(*ω*)|^2^ is the spectral density at frequency *ω*; *n*(*ω*_*β*_) is the occupation number of the *ω*_*β*_ mode, and 
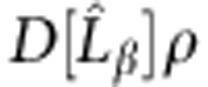
 is the Lindbladian dissipator 
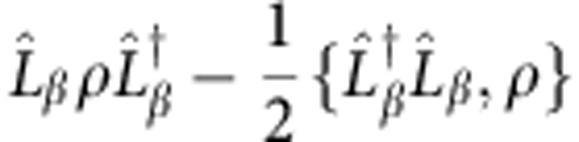
. 
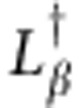
 moves the system up a Dicke ladder transition with frequency *ω*_*β*_.

Equation (7) also features unitary dynamics due to the field interaction that comprises two components: the Lamb shift, accounted for by renormalising *ω*_A_ in the system Hamiltonian *Ĥ*_S_, and the field induced dipole–dipole interaction


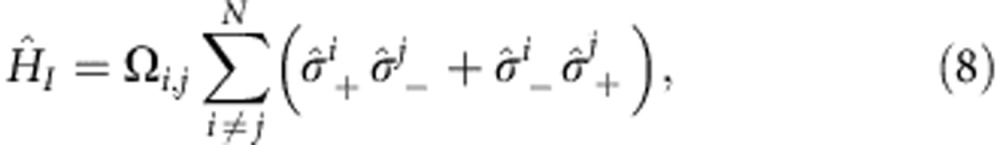


which describes energy conserving ‘hopping’ of excitons between sites mediated by virtual photon exchange. Such interactions can also be added to the system implicitly, yielding analogous results (see Supplementary Method 4). The hopping interaction strength *Ω*_*i,j*_ is given by ref. 4


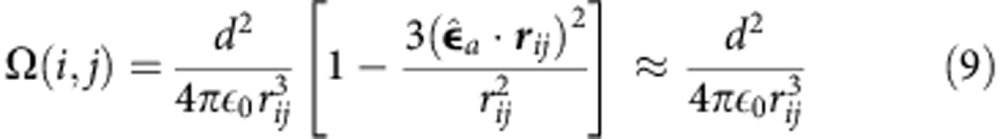


with 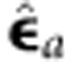 being a unit vector parallel to the direction of the dipoles. For a circular geometry with dipoles perpendicular to ***r***_*ij*_ and retaining only nearest neighbour interactions (a good approximation for larger rings since 
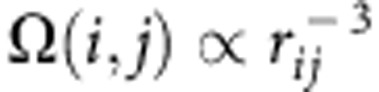
), *Ω*:=*Ω*(*i*, *i*+1) is a constant. However, note that the restriction to εnearest neighbour coupling is not a requirement; please see the Supplementary Method 3 for a full discussion. Owing to the high degree of symmetry of the ring geometry, to first order *Ĥ*_I_ does not mix the |*J,M*› eigenstates, only shifting their energies^4^ according to


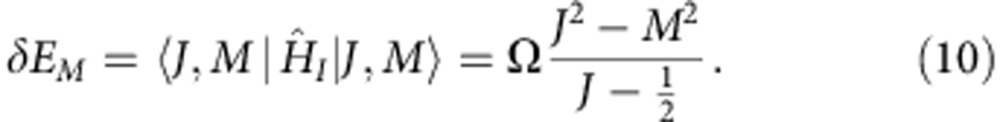


The shift of the transition frequencies is given by the difference of two adjacent levels *E*_M_−*E*_M−1_:


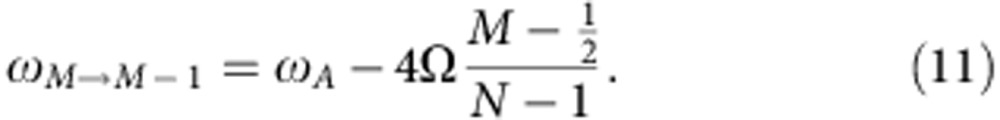


These altered frequencies break the degeneracy in the Dicke ladder where each transition now has a unique frequency. For example the transition frequency from the ground state to the first Dicke state is *ω*_−*N*/2+1→−*N*/2_=*ω*_A_−2*Ω*. Crucially, the Dicke states still represent a very good approximation of the eigenbasis of the system, yet each transition in the ladder now samples both *κ*(*ω*) and *n*(*ω*) at its own unique frequency. One might expect that the speed of the collective transitions could cause sufficient lifetime broadening to mask the shifts. However in Supplementary Method 2, we show that this is not the case.

### Transition rate engineering

**Figure 2:**
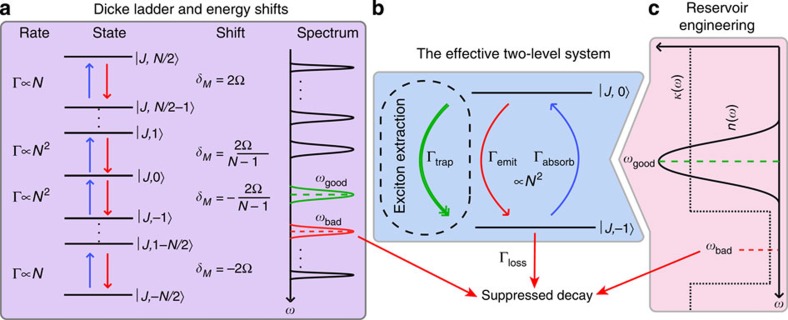
Engineering the Dicke ladder. (**a**) The ladder of Dicke states of an *N* atom system, with emission (red) and absorption (blue) processes. In the presence of interactions *Ω*≠0, the frequency shift of each transition is given by *ω*_A_+*δ*_M_. (**b**) The effective two-level system (E2LS) picture with the optional trapping process for energy extraction in the dashed box. (**c**) A scheme for using the environment to confine the ladder of states into an effective two-level system either by tailoring the spectral density *κ*(*ω*) or the mode occupation *n*(*ω*).

Our aim is to enhance transition rates at the frequency of the E2LS, which we shall call the ‘good' frequency (*ω*_0→−1_=*ω*_good_) and suppress those for transitions directly out of the E2LS at the ‘bad’ frequency (*ω*_−1→−2_=*ω*_bad_). The required type of control of the environment is known as reservoir engineering^17^; in principle we have a choice between tailoring *κ*(*ω*), *n*(*ω*) or both. Tailoring the spectral density has the advantage that it can, in theory, completely eliminate the rate of loss from our E2LS when there is no mode of the right frequency present to allow decay. This requires placing the device inside a suitably designed cavity or a photonic bandgap (PBG) crystal with a stop band at *ω*_bad_ (see Fig. 2c), where the required dimensionality of the PBG depends on the orientation of the optical dipoles. Suppression of emission rates by several orders of magnitude is then achievable with state-of-the-art systems^18^,^19^,^20^,^21^. Photonic crystal cavities can offer both enhancement of a resonant transition (*ω*_good_) and suppression of an off-resonant one (*ω*_bad_; ref. 22), making them ideal for the type of control required.

Control of *n*(*ω*) is technically easier to achieve, for example, by using filtered thermal or pseudothermal^23^,^24^ light. However, this approach has the limitation that even in the optimal control regime, where *n*(*ω*)=0 everywhere except in a narrow region around *ω*_good_, spontaneous emission will still cause loss from the E2LS.

Since both environmental control approaches rely on frequency selectivity, a sufficiently large detuning between adjacent Dicke transitions will be critical for achieving effective containment within the E2LS. Fortunately, this detuning is already within the frequency selectivity of current experimental controls for moderately sized rings, of say *N*~10: see the Supplementary Tables 1 and 2.

In practice the environmental control will never be quite perfect and our system will, over long times, inevitably evolve away from the E2LS. For example, one may only have control over *n*(*ω*) but not *κ*(*ω*), or an imperfect PBG with *κ*(*ω*_bad_)>0, and both cases lead to an exponential decay of E2LS population with the lifetime 
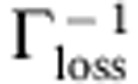
. Dephasing processes will also lead to leakage out of the fully symmetric subspace and thus shorten the effective lifetime of the E2LS. However, these imperfections need not dominate the behaviour and destroy the effect. We shall discuss the issue of sustained operation in the reinitialization section.

**Figure 3:**
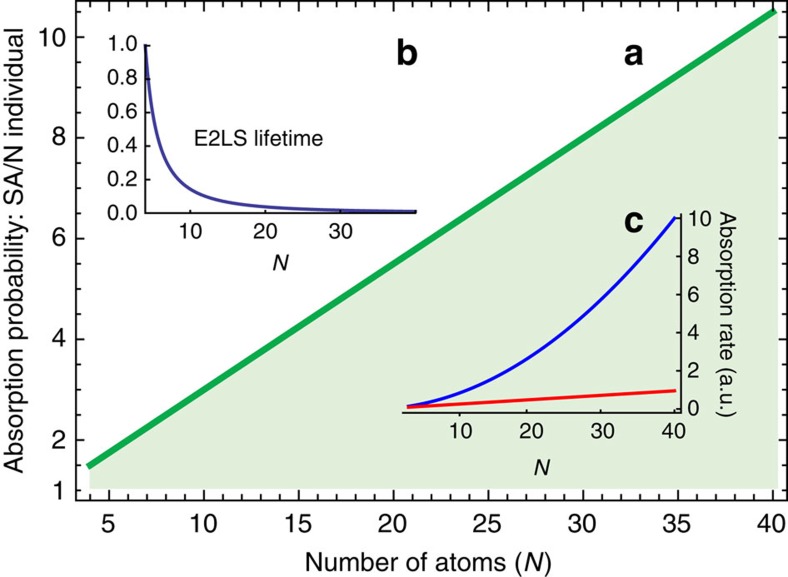
Enhanced absorption probability. (**a**) the probability of absorbing a photon within the lifetime 
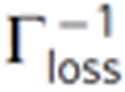
 (*N*) of the superabsorbing E2LS comprising *N* atoms, compared with that of *N* individual atoms over the same duration. The relative advantage is linear in *N* as expected, and the coloured shading indicates the quantum advantage. (**b**) lifetime of the E2LS for growing *N* relative to the four atoms case 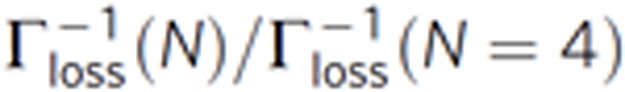. Note that the decrease in lifetime corresponds to an increasing time resolution of a superabsorbing photon detector: after initialization the system is receptive to a photon of the requisite frequency only during this time window. (**c**) absorption rate at the midpoint of the Dicke ladder (blue) and for *N* individual absorbers (red). The clearly visible *N*^2^ scaling that is typical of superradiant pulses also applies to the absorption rate.

Let us now consider the properties of the system immediately following initialization: Figure 3 shows the increased photon absorption rate of the superabsorbing E2LS compared with *N* uncorrelated atoms, Γ_−1→0_/*N*. Clearly, the probability of absorbing a photon within a given time window (up to the E2LS lifetime) is much higher in the superabsorbing configuration, providing an opportunity for photon dectection with improved sensitivity. The inset of Fig. 3 shows the lifetime of the E2LS, 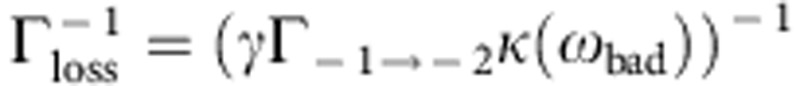, as a function of *N*, here assumed to be limited by an imperfect PBG with *κ*(*ω*_bad_)/*κ*(*ω*_good_)=1/100. For photon sensing, the reduction of the operational window with increasing *N* may even be a desirable attribute (offering time resolved detection). Generally, the system we have so far described can function as sensor as long as the temporary presence of an additional exciton can be registered, for example, through continuously monitoring the system’s charge state with a quantum point contact^25^,^26^,^27^,^28^.

### Trapping

We have detailed how to create a photon sensor using superabsorption. We can also employ the superabsorption phenomenon in the context of energy harvesting if we can meet a further requirement: a non-radiative channel to extract excitons from the upper of these two levels, turning them into useful work as depicted in the dashed box of Fig. 2b. Specifically, we require an irreversible trapping process that extracts only the excitons that are absorbed by the E2LS, and does not extract excitons from levels below the E2LS. Moreover, the trapping process competes with the re-emission of the photons at a rate proportional to *n*(*ω*_good_)+1, so that ideally it is much faster than that. Note that in this limit saturation is not an issue since absorbed photons are quickly transferred and converted, leaving the system free to absorb the next photon.

The excitons are delocalized across the ring and need to be extracted collectively to preserve the symmetry of the Dicke states. In designing this process we take inspiration from natural light harvesting systems: a ‘trap’ atom is located at the centre of the ring and symmetrically coupled via a resonant hopping interaction to all the other atoms (see Fig. 1). The corresponding trapping Hamiltonian is


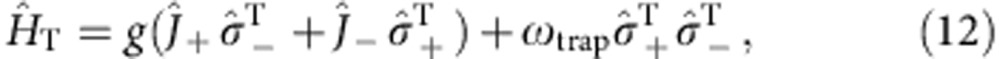


where the superscript T denotes the trap site, *g* is the strength of the coupling between the ring and the trap, and the trap’s transition frequency *ω*_trap_ ideally matches *ω*_good_. In this case the interaction is mediated by the electromagnetic field as described in the previous section, but it could have other physical origins depending on the system of interest. Once an exciton has moved to the trap site we assume that it is then removed into the wider environment by a process which irreversibly absorbs its energy. We note that more exotic and potentially far more efficient trapping implementations can be envisioned, such as, for example, a reservoir of excitons with an effective ‘Fermi level’ capable of accepting excitons only above the energy level *E*_−1_. However, at present our aim is to focus on the simplest system capable of exhibiting enhanced photon energy harvesting by superabsorption.

The above trapping process is adequately described phenomenologically (see Supplementary Method 6, Supplementary Figs 1 & 2) as collective exciton extraction from the midpoint (*M*=0) by adding the dissipator 
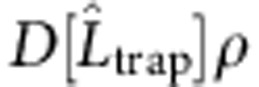
 to the righthand side of equation (7) with 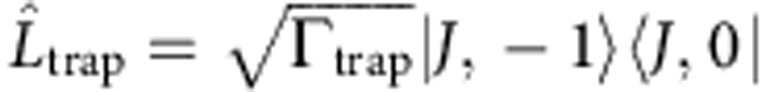, and where Γ_trap_ is the rate of the trapping process. The rate of exciton extraction *I*_trap_ is then simply given by the population of the trapping level multiplied by the trapping rate:


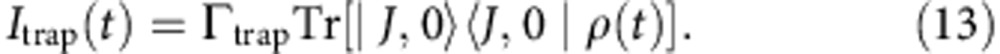


Consider an ideal E2LS realized by a PBG completely blocking *ω*_bad_, that is, a vanishing Γ_loss_:=*κ*(*ω*_bad_)(*n*(*ω*_bad_)+1)Γ_−1→−2_. Assuming a faster trapping than emission rate, Γ_trap_>>Γ_emit_:=*κ*(*ω*_good_)(*n*(*ω*_good_)+1)Γ_0→−1_, our figure of merit *I*_trap_ matches the absorption rate Γ_absorb_:=*κ*(*ω*_good_)*n*(*ω*_good_)Γ_−1→0_ for all *t*:


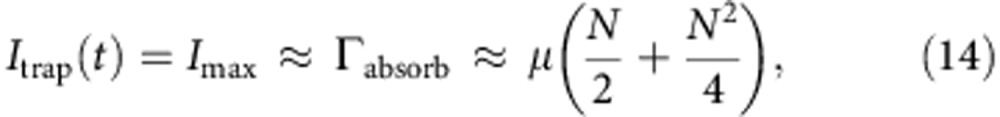


where *μ*=*γκ*(*ω*_good_)*n*(*ω*_good_). It is clear from this equation that under these conditions we achieve a superlinear scaling of the exciton current flowing out of the superabsorber. Trapping processes like the one described here have been demonstrated experimentally and meet the requirement Γ_trap_>>Γ_emit_, see Supplementary Method 6.

**Figure 4:**
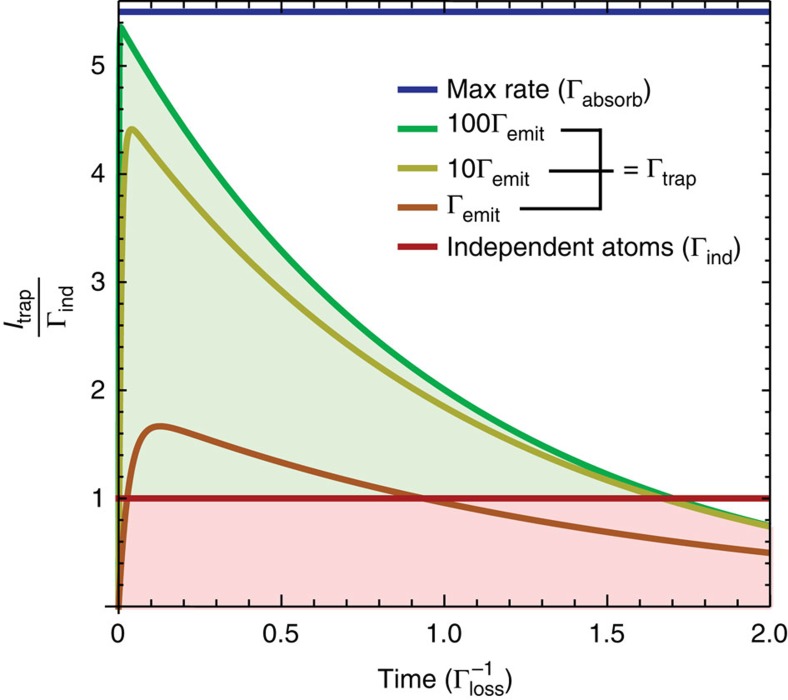
A superabsorption cycle. Superabsorption of the effective two-level system indicated in Fig. 2. The green shading indicates the superabsorption region, the red when the extraction rate is below what could be extracted from uncorrelated atoms; both are for a system of 20 atoms and mode occupancy *n*(*ω*_good_)=10. The maximum extraction possible from independent atoms (Γ_ind_=*n*(*ω*_good_)*N*_*γ*_) is used for comparison.

The inevitable loss out of the E2LS entails an exponential decay of *I*_trap_(*t*) with the lifetime 
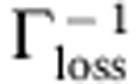
, as shown in Fig. 4. The initial net superabsorption rate far exceeds that possible from uncorrelated atoms, however it is only a transient effect and the system needs to be reinitialized periodically to maintain its advantage. This aspect will be discussed in the next section.

We have detailed the case where a PBG is used to increase the lifetime of the E2LS. If instead intense filtered thermal light is used to ensure *n*(*ω*_good_)>>1, then many absorption-trapping cycles can take place before a spontaneous emission event happens. This set-up would enable quantum-enhanced light-based power transmission, where a large number of photons need to be harvested quickly in a confined area.

### Reinitialization

Reinitialization could be achieved by exploiting a chirped pulse of laser light to re-excite the system, or through a temporary reversal of the trapping process. In practice there will be an energy cost associated with reinitialization but, as we show below, in all but the most severe cases this cost is more than offset by the faster photon to exciton conversion rate during the transient superabsorption period. Furthermore, the frequency with which one has to reinitialize does not have a fundamental lower bound, it is limited only by the quality of the control one can apply.

Perhaps the most elegant way of implementing the reinitialization step (short of self-initialization, see below) would make use of quantum feedback control^29^: The superabsorption enhancement is derived from coherence between states that all possess the same number of excitons. Therefore, the number of excitons could be continually monitored (for example, by a quantum point contact or by monitoring fluorescence of a probe field tuned to a level or two below the desired manifold) without destroying the desired effect. A suitably designed feedback system could then feed in an excitation only when a loss event occurs, providing optimal efficiency


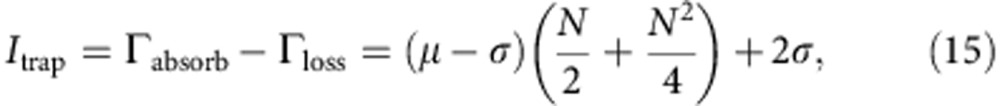


where *σ*=*γκ*(*ω*_bad_)(1+*n*(*ω*_bad_)). Provided *μ*>*σ* superabsorption will occur, and for *σ*=0, we recover the theoretical maximum of the idealized case in equation (14).

**Figure 5:**
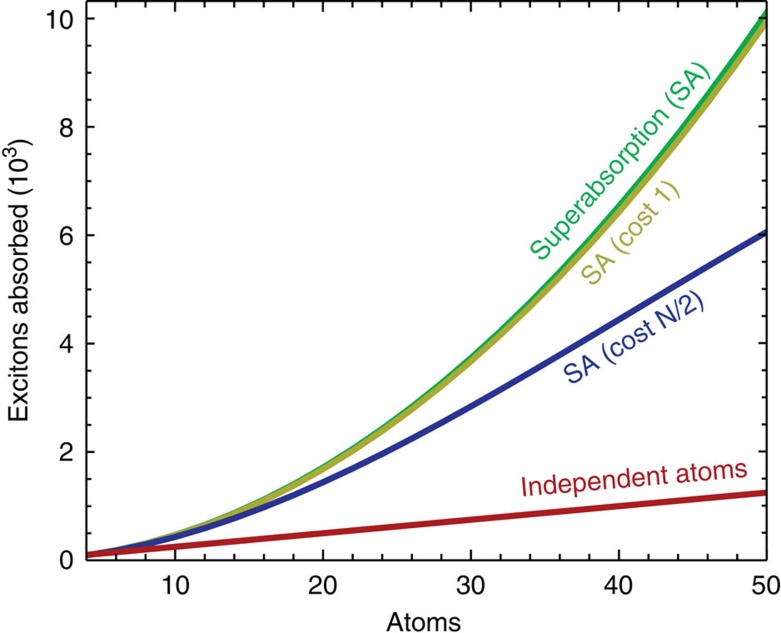
Superlinear exciton absorption. The total number of excitons absorbed within the common reference time 
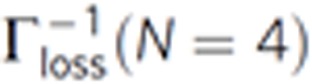
 as a function of the number of atoms *N*. The coloured curves represent the reinitialization cost models described in the main text, and the red line shows the maximum extracted from independent atoms for comparison. The scaling is superlinear in all coupled atom cases, approximately following the ideal *N*^2^ law (green), except for large *N* in the pessimistic cost model of full reinitialization (blue). If quantum feedback control enables the replacement of a single exciton as soon as a loss event has happened, then the nearly quadratic scaling persists up to an arbitrary number of atoms (olive).

A far simpler reinitialization scheme would only require periodic reinitialization following a fixed time interval, and does away with the need for feedback control. To account for the relative cost of such reinitialization, we need to quantify the total number of excitons absorbed in a given time. Let us fix the time at which reinitialization is performed to the natural lifetime of the E2LS, 
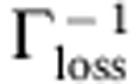
. Integrating the trapping rate *I*_trap_(*t*) over one lifetime and subtracting the reinitialization cost gives a fair measure of the number of excitons the system has absorbed within the given time. We can then consider the extreme limits of the reinitialization cost, from simply replacing a single lost exciton, to having to replace all of the *N*/2 excitons that make up the superabsorbing state. A larger system requires more frequent reinitializations, since its loss rate is also enhanced by the system size. However, the bias in favour of absorption created by the environmental control is sufficient to ensure this does not negate the superabsorption process. Figure 5 shows how the number of excitons absorbed in a given time scales with the number of atoms, and for all cost models we find a superlinear scaling.

## Discussion

We have shown that the absorptive analogue of quantum superradiance can be engineered in structures with suitably symmetric interactions. We have provided an intuitive explanation of this many-body light–matter effect by introducing an E2LS. Despite its simplicity this analytic model can provide highly accurate predictions, as we have validated through the extensive exact numerical calculations that are summarized in the Methods section, see Supplementary Method 5 and Supplementary Fig. 3. As we have already discussed, absorbing light beyond the limits of classical physics raises prospects for at least two new types of technology, and superabsorption could be realized in a broad range of candidate systems.

The foremost application of the phenomenon may be in the context of optical or microwave sensors, either in future cameras or for scientific instruments. In addition to the obvious merits of being sensitive to low light levels, the frequency specificity of the superabsorber may be a desirable attribute. The small size of the ring structure and collective ‘antenna array’ could lead to high spatial and angular resolution, and the fact that the superabsorber is (re)initialized into its fully receptive state by an excitation pulse allows detection events to be confined into a narrowly defined time window. Note that for sensing applications the cost of (re)initialization is likely unimportant, and a trapping mechanism is not required if the number of excitons in the system can be monitored differently, for example, with a quantum point contact.

Light harvesting technologies represent another potential application, and indeed our Fig. 5 indicates that one can obtain a net increase in the number of exctions absorbed compared with conventional systems even allowing for the energy cost of sustaining the superabsorbing state. The technique would be particularly suited to wireless power transfer using narrowband light, for example, for remote sensors or biologically implanted devices, where wired electrical power is impractical. For solar light harvesting a given superabsorber can only achieve optimal performance for a specific frequency range; however, one could engineer a range of different systems to jointly cover the solar spectrum.

There are multiple candidate systems for engineering the above applications. Molecular rings have the advantage of featuring a natural symmetry and intrinsically low levels of disorder. Taking *Ω*=350 cm^−1^ as appropriate for a B850 ring (ref. 30) with eight atoms produces transition wavelength shifts exceeding 6 nm, and a wavelength selectivity on the scale of nanometres is readily available with current laser and cavity linewidths. Of course, the dipole alignment of the B850 ring is not optimized for this purpose. However, complex ring structures can be designed and synthesized artificially (for example, porphyrin rings^31^) and this route should enable far superior properties. Self-assembly into much larger molecular *J* or *H* aggregates with established superradiant properties^32^,^33^ may provide further opportunities. Alternatively, superradiance, long-range interactions and optical control have been demonstrated in quantum dots^2^,^34^, and there has been recent progress in synthesizing ring-like clusters with high spectral and spatial order^35^. Further, suppression of the local density of optical states by two orders of magnitude at specific frequencies has been demonstrated in an appropriate semiconductor photonic crystal environment^18^. For typical parameters of those systems that would be consistent with the requirements for superabsorption see the Supplementary Discussion 1.

To demonstrate the effect of superabsorption (that is, sustained confinement into an E2LS with enhanced absorption and emission rates) as an instance of an engineered physical phenomenon, several additional possibilities present themselves. For example, circuit QED experiments possess long coherence times and have already demonstrated sub and superradiant effects^36^,^37^, as well as tuneable cross Lamb shifts^38^ and recent three-dimensional structures^39^ provide further flexibility. Bose–Einstein Condensates offer similar properties but with much larger numbers of atoms^40^,^41^. Dissipative Dicke model studies with nonlinear atom-photon interaction can enable a steady-state at the midpoint of the Dicke ladder (*M*=0; refs 42, 43), which may provide a route to self-initialising superabsorbing systems.

## Methods

### Collective master equation

The master equation (7) is an *N* atom generalization of the standard quantum optical master equation; we give the full derivation in the Supplementary Method 1. In particular, it assumes that all *N* atoms are spatially indistinguishable due to occupying a volume with linear dimensions much smaller than the relevant wavelength of light. In addition, interactions between atoms must respect certain symmetry requirements to only shift the Dicke states to first order (for example, as is exemplified by equation (10)). However, as we also discuss in Supplementary Method 5—and verify with numerical calculations—superlinear scaling of the absorption rate with the number of atoms remains possible beyond a first order perturbative treatment of suitably symmetrical interactions.

### Numerical calculations

The E2LS model reduces the complexity of the problem and makes it analytically tractable. To verify this approach we have compared it with two different independent numerical models. Supplementary Fig. 3 shows excellent agreement between the E2LS model and the Monte Carlo simulations of the master equation (7). In Supplementary Figs 1 and 2, we extend the model further by incorporating an explicit trap site and allow coherent transfer from the ring to the trap, as described in the Trapping Section, showing that superabsorption is still realized in that case and that the E2LS model still provides a good description of the behaviour. This model uses a generalized master equation solved numerically.

### Imperfections

Any real physical system used to demonstrate superabsorption or indeed superradiance, will have imperfections such as slightly varying frequencies for each atom, or a deviation away from perfect ring symmetry. In essence all such imperfections in superradiance are alike; they diminish the collective effect because they lead to the emission of distinguishable photons. It might therefore be a concern that these collective effects could only be realized in the ideal case. However, superradiant effects of molecular aggregates with a spatial extent smaller than the wavelength of light are known to possess a certain degree of robustness against inhomogenous broadening^44^, dephasing processes^45^ and exciton phonon coupling^46^. This is because the increased transition rates produced by superradiance serve to counterbalance the effect of disorder: the faster rate broadens the natural linewidth of the transitions, effectively preventing the introduction of distinguishability from the disorder. Intuitively, we expect a superabsorption advantage to be achievable whenever an imperfect system is still capable of displaying superradiant behaviour (of course with the additional requirement that the energy shifts of adjacent decay process are resolvable). In Supplementary Method 5 and Supplementary Figs 4 and 5, we model realistic imperfections by considering static energy disorder and show that superabsorption can still be realized in the presence of disorder.

## Author contributions

All authors designed the protocol, analyzed the results and discussed the manuscript. K.D.B.H., B.W.L., and E.M.G. performed the calculations. K.D.B.H., S.C.B., B.W.L. and E.M.G. wrote the manuscript.

## Additional information

**How to cite this article:** Higgins, K. D. B. *et al.* Superabsorption of light via quantum engineering. *Nat. Commun.* 5:4705 doi: 10.1038/ncomms5705 (2014).

## Supplementary Material

Supplementary Information Supplementary Figures 1-5, Supplementary Tables 1-2, Supplementary Discussion, Supplementary Methods 1-6 and Supplementary References

## References

[b1] Dicke R. H. Coherence in spontaneous radiation processes. Phys. Rev. 93, 99–110 (1954).

[b2] Scheibner M. *et al.* Superradiance of quantum dots. Nat. Phys. 3, 106–110 (2007).

[b3] Wang H., Zheng X., Zhao F., Gao Z. & Yu Z. Superradiance of high density frenkel excitons at room temperature. Phys. Rev. Lett. 74, 4079–4082 (1995). 10058407 10.1103/PhysRevLett.74.4079

[b4] Gross M. & Haroche S. Superradiance: an essay on the theory of collective spontaneous emission. Phys. Rep. 93, 301–396 (1982).

[b5] Brandes T. Coherent and collective quantum optical effects in mesoscopic systems. Phys. Rep. 408, 315–474 (2005).

[b6] Monshouwer R., Abrahamsson M., van Mourik F. & van Grondelle R. Superradiance and exciton delocalization in bacterial photosynthetic light-harvesting systems. J. Phys. Chem. B 101, 7241–7248 (1997).

[b7] Bohnet J. G. *et al.* A steady-state superradiant laser with less than one intracavity photon. Nature 484, 78–81 (2012). 22481360 10.1038/nature10920

[b8] van Putten M. H. P. M. Superradiance in a torus magnetosphere around a black hole. Science 284, 115–118 (1999). 10102805 10.1126/science.284.5411.115

[b9] Blankenship R. E. Molecular Mechanisms of Photosynthesis 1st edn Blackwell Science (2002).

[b10] Dong H., Xu D.-Z., Huang J.-F. & Sun C.-P. Coherent excitation transfer via the dark-state channel in a bionic system. Light Sci. Appl. 1, e2 (2012).

[b11] Chen X.-W., Sandoghdar V. & Agio M. Coherent interaction of light with a metallic structure coupled to a single quantum emitter: from superabsorption to cloaking. Phys. Rev. Lett. 110, 153605 (2013). 25167268 10.1103/PhysRevLett.110.153605

[b12] Wan W. *et al.* Time-reversed lasing and interferometric control of absorption. Science 331, 889–892 (2011). 21330539 10.1126/science.1200735

[b13] Haake F., Kolobov M. I., Fabre C., Giacobino E. & Reynaud S. Superradiant laser. Phys. Rev. Lett. 71, 995–998 (1993). 10055422 10.1103/PhysRevLett.71.995

[b14] Meiser D. & Holland M. J. Steady-state superradiance with alkaline-earth-metal atoms. Phys. Rev. A 81, 033847–033850 (2010).

[b15] Auffèves A., Gerace D., Portolan S., Drezet A. & Santos M. F. Few emitters in a cavity: from cooperative emission to individualization. New J. Phys. 13, 093020 (2011).

[b16] Coffey B. & Friedberg R. Effect of short-range coulomb interaction on cooperative spontaneous emission. Phys. Rev. A 17, 1033–1048 (1978).

[b17] Pechen A. & Rabitz H. Teaching the environment to control quantum systems. Phys. Rev. A 73, 062102 (2006).

[b18] Wang Q., Stobbe S. & Lodahl P. Mapping the local density of optical states of a photonic crystal with single quantum dots. Phys. Rev. Lett. 107, 167404 (2011). 22107427 10.1103/PhysRevLett.107.167404

[b19] Noda S., Fujita M. & Asano T. Spontaneous-emission control by photonic crystals and nanocavities. Nat. Photon. 1, 449–458 (2007).

[b20] Leistikow M. *et al.* Inhibited spontaneous emission of quantum dots observed in a 3d photonic band gap. Phys. Rev. Lett. 107, 193903 (2011). 22181609 10.1103/PhysRevLett.107.193903

[b21] Frölich A., Fischer J., Zebrowski T., Busch K. & Wegener M. Titania woodpiles with complete three-dimensional photonic bandgaps in the visible. Adv. Mater. 25, 3588–3592 (2013). 23703892 10.1002/adma.201300896

[b22] Englund D. *et al.* Controlling the spontaneous emission rate of single quantum dots in a two-dimensional photonic crystal. Phys. Rev. Lett. 95, 013904 (2005). 16090618 10.1103/PhysRevLett.95.013904

[b23] Martienssen W. & Spiller E. Coherence and fluctuations in light beams. Am. J. Phys. 32, 919–926 (1964).

[b24] Goodman J. W. Statistical Optics 567Wiley-Interscience (1985).

[b25] Gurvitz S. A. Measurements with a noninvasive detector and dephasing mechanism. Phys. Rev. B. 56, 15215 (1997).

[b26] Goan H.-S. Continuous measurements of electron tunneling through a quantum dot by a quantum point contact. Bull. Am. Phys. Soc. 56, 513 (2011).

[b27] Elzerman J. *et al.* Few-electron quantum dot circuit with integrated charge read out. Phys. Rev. B 67, 161308 (2003).

[b28] Petta J., Johnson A., Marcus C., Hanson M. & Gossard A. Manipulation of a single charge in a double quantum dot. Phys. Rev. Lett. 93, 186802 (2004). 15525191 10.1103/PhysRevLett.93.186802

[b29] Wiseman H. M. & Milburn G. J. Quantum Measurement and Control 1st edn Cambridge University Press (2010).

[b30] van Oijen A. M., Ketelaars M., Köhler J., Aartsma T. J. & Schmidt J. Unraveling the electronic structure of individual photosynthetic pigment-protein complexes. Science 285, 400–402 (1999). 10411501 10.1126/science.285.5426.400

[b31] O'Sullivan M. C. *et al.* Vernier templating and synthesis of a 12-porphyrin nano-ring. Nature 469, 72–75 (2011). 21209660 10.1038/nature09683

[b32] McHale J. L. Hierarchal structure of light-harvesting porphyrin aggregates. J. Aggregates 2, 77 (2012). 10.1039/c2cp23362b 22241160

[b33] Saikin S. K., Alexander E., Stéphanie V. & Alán A.-G. Photonics meets excitonics: natural and artificial molecular aggregates. Nanophotonics 2, 21 (2013).

[b34] Unold T., Mueller K., Lienau C., Elsaesser T. & Wieck A. D. Optical control of excitons in a pair of quantum dots coupled by the dipole-dipole interaction. Phys. Rev. Lett. 94, 137404 (2005). 15904035 10.1103/PhysRevLett.94.137404

[b35] Creasey M., Lee J.-H., Wang Z., Salamo G. J. & Li X. Self-assembled ingaas quantum dot clusters with controlled spatial and spectral properties. Nano Lett. 12, 5169–5174 (2012). 22992172 10.1021/nl3021736

[b36] Fink J. M. *et al.* Dressed collective qubit states and the tavis-cummings model in circuit qed. Phys. Rev. Lett. 103, 083601 (2009). 19792728 10.1103/PhysRevLett.103.083601

[b37] Filipp S., van Loo A. F., Baur M., Steffen L. & Wallraff A. Preparation of subradiant states using local qubit control in circuit QED. Phys. Rev. A 84, 061805 (2011).

[b38] Lalumière K. *et al.* Tuning from coherent interaction to super-and subradiance with artificial atoms in a 1d waveguide. APS Meeting Abstr. 1, 25012 (2013).

[b39] Paik H. *et al.* Observation of high coherence in josephson junction qubits measured in a three-dimensional circuit QED architecture. Phys. Rev. Lett. 107, 240501 (2011). 22242979 10.1103/PhysRevLett.107.240501

[b40] Colombe Y. *et al.* Strong atom-field coupling for bose-einstein condensates in an optical cavity on a chip. Nature 450, 272–276 (2007). 17994094 10.1038/nature06331

[b41] Nagy D., Kónya G., Szirmai G. & Domokos P. Dicke-model phase transition in the quantum motion of a bose-einstein condensate in an optical cavity. Phys. Rev. Lett. 104, 130401 (2010). 20481867 10.1103/PhysRevLett.104.130401

[b42] Dimer F., Estienne B., Parkins A. & Carmichael H. Proposed realization of the dicke-model quantum phase transition in an optical cavity qed system. Phys. Rev. A 75, 013804 (2007).

[b43] Grimsmo A. L. & Parkins S. Cavity-qed simulation of qubit-oscillator dynamics in the ultrastrong-coupling regime. Phys. Rev. A 87, 033814 (2013).

[b44] Spano F. C. & Mukamel S. Superradiance in molecular aggregates. J. Chem. Phys. 91, 683–700 (1989).

[b45] Grad J., Hernandez G. & Mukamel S. Radiative decay and energy transfer in molecular aggregates: the role of intermolecular dephasing. Phys. Rev. A 37, 3835–3846 (1988). 9899496 10.1103/physreva.37.3835

[b46] Spano F. C. Fermion excited states in one-dimensional molecular aggregates with site disorder: nonlinear optical response. Phys. Rev. Lett. 67, 3424–3427 (1991). 10044730 10.1103/PhysRevLett.67.3424

