# Supplementary material for: Superabsorption of light via quantum engineering
Source: Nat Commun. 2014 Aug 22;5:4705. doi: 10.1038/ncomms5705 (PMC4143938; doi:10.1038/ncomms5705)
Supplement: Supplementary Information — Supplementary Figures 1-5, Supplementary Tables 1-2, Supplementary Discussion, Supplementary Methods 1-6 and Supplementary References [file ncomms5705-s1.pdf]

Supplementary Information: Superabsorption of light via quantum engineering

I. SUPPLEMENTARY FIGURES

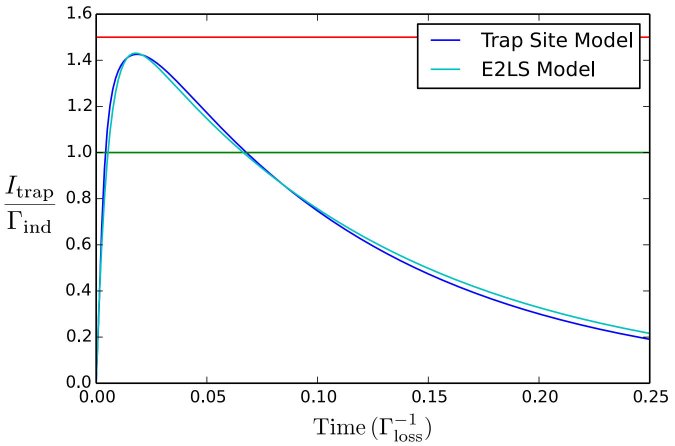

Supplementary Figure 1: Comparison between the full trapping model discussed in Supplementary Method 6 modelled numerically using the technique described in Supplementary Method 5 and the effective two level system model with adjusted rates. Parameters are: $d = 0.1, \gamma = 10^{-4}, g = 1$, and $\Gamma_{\rm trap} = 4g$. In the phenomenological case $\Gamma_{\rm trap}$ takes the same value, whereas the effective $\Gamma_{\rm loss}$ rate was increased by a factor of five to account for additional leakage out of the E2LS due to detuned exciton extraction processes (see Supplementary Fig. 2). Note that the shape of the E2LS curve can be made to look more similar to the full model by adjusting its $\Gamma_{\rm trap}$ and $\Gamma_{\rm loss}$ rates, but here we have chosen values giving rise to a similar peak height and enhancement area, indicative of a comparable collective advantage over one superabsorption lifetime cycle.

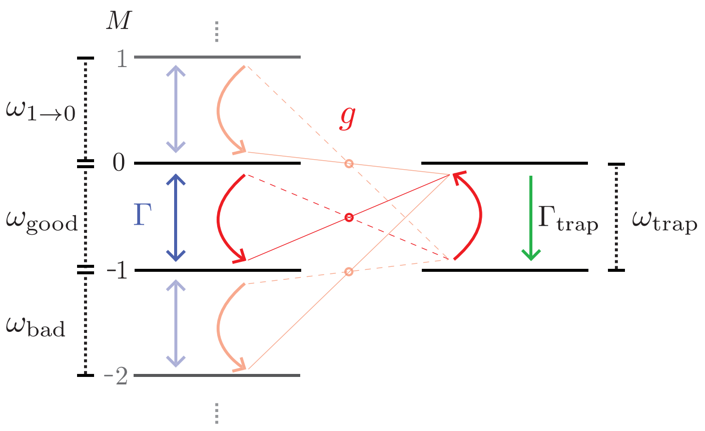

Supplementary Figure 2: A trap site is connected to the Dicke ladder. The trap's transition frequency ideally matches that of the 'good' transition $\omega_{\rm trap} \approx \omega_{\rm good}$, and it is coupled to the Dicke transitions via a flip-flop interaction of strength g. This gives rise to 'see-saw' like oscillations between Dicke and trap transitions, but only the desired transition is resonant, all others are detuned and thus suppressed. To ensure that excitons hopping to the trap site are irreversibly removed instead of 'see-sawing' back and forth indefinitely, the trap is incoherently emptied at a rate $\Gamma_{\rm trap}$. As long as $\Gamma_{\rm trap}$ is not so large that the trap transition experiences significant lifetime broadening approaching $|\omega_{\rm good} - \omega_{\rm bad}|$, and also assuming $|\omega_{\rm good} - \omega_{\rm bad}| > g$, the exciton extraction from the 'bad' transition in particular can be suppressed. The blue double-headed arrows indicate the (enhanced) optical emission and absorption processes from the main text.

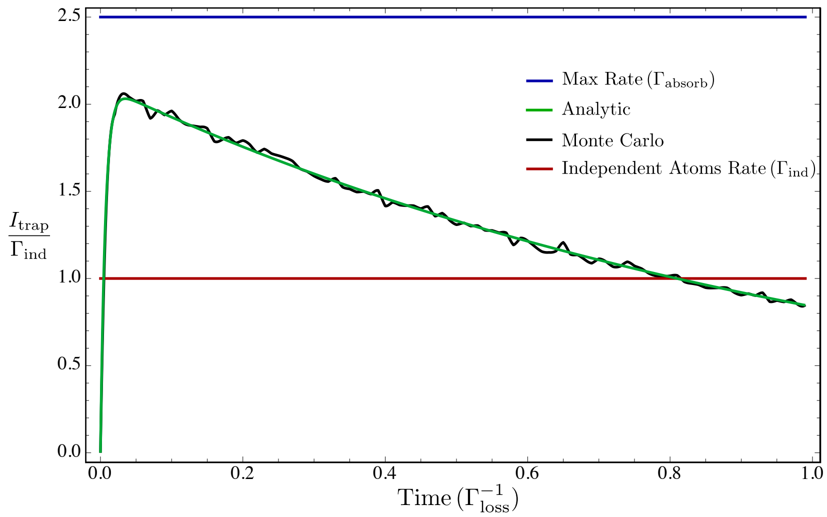

Supplementary Figure 3: Comparison of the Effective Two Level System (E2LS) model with numerical results from Monte Carlo simulations done using the Quantum Optics Toolbox in Python (QuTip)[1]. Parameters: $N = 8$, $N(\omega_{\mathrm{good}}) = 10$, $\Gamma_T = 10\,\Gamma_E$,$\gamma = 1$, trajectories 100,000.

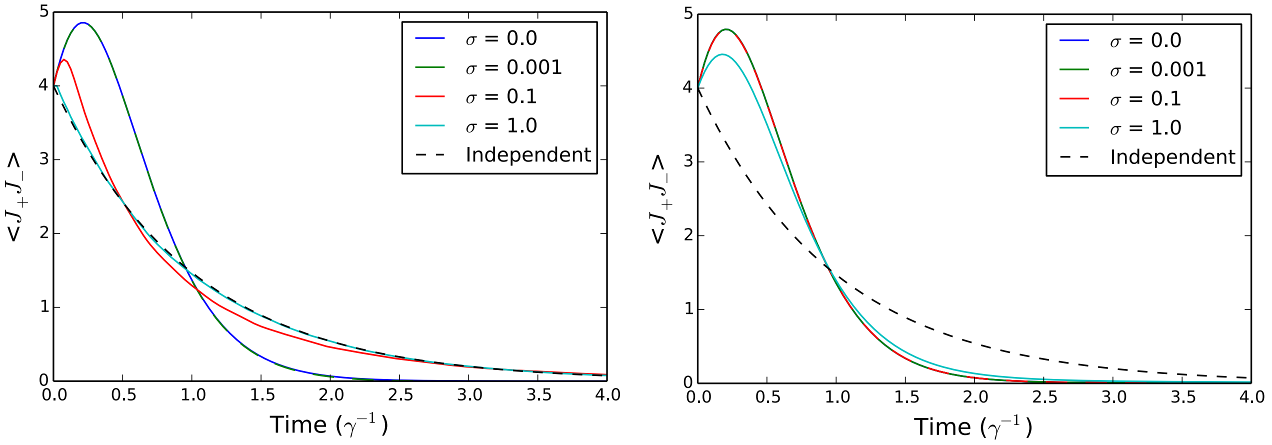

Supplementary Figure 4: Left: The effect of increasing disorder (modelled as a Gaussian distribution with standard deviation σ) on a superradiant system without interactions ($\Omega = 0$). Right: The effect of increasing disorder (σ) on a superradiant system with hopping interaction strength $\Omega = -1$. Shared parameters: $\omega_A = 10, d = 1, \gamma = 0.01, N = 4$. Without interactions, the relevant energy scale characterising the transition from collective to independent emission is given by σ/γ. By contrast, when interactions are included the system eigenstates are intrinsically delocalised and the relevant energy scale becomes σ/Ω, leading to a significant increase in robustness against disorder.

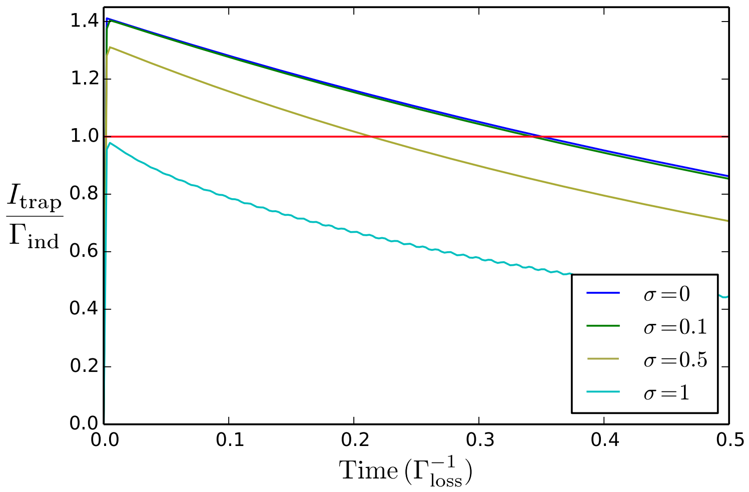

Supplementary Figure 5: This figure shows that modest amounts of disorder will not significantly the superabsorption effect. Parameters: $\omega_A = 10$, d=1, $\gamma = 0.01$, N=4, $N(\omega_{\rm good}) = 1$, $\kappa(\omega_{\rm bad}) = 0.1\gamma$.

II. SUPPLEMENTARY TABLES

Type	E (eV)	γ^{-1} (ns)	d (D)	r_{ij} (nm)	Ω (eV)	σ (meV)	$\lambda_{\rm good} - \lambda_{\rm bad}\|_{N=10}$ (nm)
Quantum Dot (Förster)	1-2	$0.1 - 1$	20-100	10-50	0.001	$\lesssim 10$	< 1
Molecular Ring (B850)	1-2	1	5	$\lesssim 1$	0.05	$\lesssim 20$	~ 10
J-aggregate	2-3	$0.05 - 1$	10-15	$\lesssim 1$	0.1-0.2	$5 - 50$	~ 45

Supplementary Table I: Materials parameters comparison: Order of magnitude estimates of the relevant parameters for the superabsorption effect for different systems. Values are taken from the literature, for supporting references see Supplementary Discussion 1.

Type	λ (nm)	$\Delta\lambda$ (nm)	Q	P_F (theory)	P_F (experiment)	$\Gamma^*_{\rm absorb}$	$\Gamma^*_{\rm loss}$
Optical Microcavity[2]	637	$\lesssim 0.1$	10^4	70	20	P_F	~ 1
Photonic Crystal Cavity[3–7]	$900 - 1500$	$\lesssim 0.05$	$10^3 - 10^6$	$10^2 - 10^4$	$5 - 147$	P_F	~ 1
Photonic Bandgap Crystals[8–11]	$400 - 1500$	n/a	n/a	n/a	n/a	~ 1	$0.01 - 0.1$
Concentrated filtered sunlight[12]	~ 650	~ 5	n/a	n/a	n/a	$\sim 10^4$	~ 1

Supplementary Table II: Materials parameters comparison for proposed control mechanisms. $\Gamma^*_{\rm absorb}$ and $\Gamma^*_{\rm loss}$ are the enhancement or suppression relative to the respective rate in the absence of a cavity or photonic band gap environment and P_F is the Purcell factor defined in Eq. (1).

III. SUPPLEMENTARY DISCUSSION 1: MATERIAL PARAMETERS FOR REAL SYSTEMS

A. System

In this section we consider the parameters for various physical systems that could potentially demonstrate the superabsorption effect. In Supplementary Table I we summarise key materials parameters for this system. Note that we use wavelength rather than frequency to characterise the good and bad transitions here, since these are more often quoted; $\omega = 2\pi c/n\lambda$, with n the material refractive index.

Quantum dots are a good candidate for their large transition dipole moments, continually improving spectral uniformity (low σ) and the recent progress in synthesising highly ordered rings and arrays[13]. The main challenge facing this implementation is the relatively weak dipole-dipole interactions (Ω) that have thus far been observed (≈ 0.01 meV), although an order of magnitude improvement in this should be easily obtainable[14]. Furthermore, the interaction need not be of the field-induced character this Article focuses on, chosen largely for the sake of simplicity. Any physical mechanism leading to an exciton number dependent shift of the Dicke states would suffice. For instance, in quantum dots the Coulomb interaction is known to be stronger (~ 10 meV)[15]. In general, engineering the strength and the scalability of dot to dot interactions is a key focus for the field, particularly for implementations of quantum information processing tasks. Progress in this area therefore seems very likely in the near future.

Molecular rings have the obvious advantage of possessing the required symmetry and the very close separation between sites, leading to large Ω. The values in the table correspond to the natural photosynthetic ring structure B850. It should be noted that lower disorder would be expected from artificially synthesised rings lacking the protein manifold found in natural systems. Furthermore, the dipole alignment of B850 (in plane) is least favourable for interactions with the field (B850 plays a storage and transfer role in photosynthesis) and therefore could be expected to increase by a factor of two if the dipoles were optimally oriented. Artificial porphyrin rings could alleviate both of these problems[16].

J-aggregates constitute a particularly promising candidate, having both highly delocalised excitations and very strong interactions between monomers[17]. Their interactions are sufficiently strong to overcome the typical values for disorder[18]. Collective effects such as superradiance and line narrowing have long been demonstrated in these systems. It is also possible to control geometry of J-aggregates chemically and deposit them on surfaces[19]. Integrating these structures with quantum control such as photonic bandgap crystals and optical microcavities is a new and developing area at the confluence of several fields. The objective of this effort is primarily the the study of exciton transport

in photosynthesis, but at the same time, these systems provide an extremely promising platform for demonstrating superabsorption.

B. Control

In the main text we describe three different proposals for implementing the required environmental control over the system: optical cavities, photonic bandgap crystals and filtered light. In Supplementary Table II we gather the relevant material parameters from the literature, again using wavelength rather than frequency units; note that $\Delta\omega/\omega = \Delta\lambda/\lambda$.

The particular figures of merit are Γ^*_{absorb} and Γ^*_{loss}, which describe the relative suppression or enhancement of the 'good' and 'bad' transitions, respectively. In the case of cavities these are given by the Purcell factor[20], which describes enhancement of the rate of emission caused by coupling to a resonant cavity mode:

$$P_F = \frac{3}{4\pi^2}\left(\frac{\lambda_c}{n}\right)^3 \frac{Q}{V_m}\,, \tag{1}$$

where λ_c is the wavelength of the cavity, n is its refractive index, $Q = \omega_c/\Delta\omega$ its quality factor, and V_m its mode volume. For the results in the main text we allowed either a tenfold enhancement or suppression of Γ_{absorb} or Γ_{loss}, respectively. Supplementary Table II shows that current experimental systems can easily surpass these requirements, assuming a typical separation of $\lambda_{\text{good}} - \lambda_{\text{bad}} > 10$ nm. Crucially, the structures listed here also remain large enough to accommodate our proposed absorber. For instance, a molecular ring with $N = 15$ would have a diameter of only ~ 5 nm. It is interesting to note that 2D and 3D photonic crystal cavities offer both enhancement of a resonant transition Γ_{absorb} and suppression of a non-resonant one Γ_{loss}[5], making them ideal for the type of control required.

C. Trapping

The trapping process is key for the application of superabsorption to light harvesting. Here the requirement is that the extraction process is fast enough to beat the rival emission process Γ_{emit}, which itself may be enhanced. Researchers concerned with creating new paradigms for solar cells by connecting quantum dots and J-aggregates (with their favourable and flexible absorption properties) to nanowires and bulk semiconductor have already been successful in realising fast and irreversible exciton extraction[21,22]. The primary mechanism used for this purpose is Förster transfer, which is well known in both natural molecular structures like photosynthetic antennae and also artificial systems such as quantum dots. Förster transfer causes extremely fast exciton transitions (fs-ps) between monomers separated by nanometer distances. This mechanism for exciton extraction has been studied both theoretically and verified experimentally. Encouragingly, it has been shown to be capable of exceeding the reemission rate by up to three orders of magnitude[23,24].

IV. SUPPLEMENTARY METHODS

V. SUPPLEMENTARY METHOD 1: MASTER EQUATION DERIVATION

In the following we sketch the derivation of the master equation (7) from the main text. Following the general procedure of Ref.[25], we generalise from a vacuum environment to one with a population distribution and structured spectral density. This allows for the presence of the superabsorption term and introduces some additional complexities.

We consider the interaction picture with respect to $\hat{H}_S$ [Eq. (1) of the main text] and the free Hamiltonian of the electromagnetic field. After performing the standard Born-Markov approximation and tracing over the environment $\mathcal{E}$, the starting point for our derivation is[26] ($\hbar = 1$):

$$\frac{d}{dt}\tilde{\rho}_{\mathcal{S}}(t) = -i\int_0^\infty dt' \text{Tr}_{\mathcal{E}}[\tilde{H}_L(t), [\tilde{H}_L(t-t'), \tilde{\rho}_{\mathcal{S}}(t) \otimes \tilde{\rho}_{\mathcal{E}}] + \text{h.c.}\,, \tag{2}$$

where $\tilde{\rho}_{\mathcal{S}}(t)$ is the reduced interaction picture density matrix and $\tilde{H}_L(t)$ denotes the interaction picture representation of the system-light-interaction Hamiltonian $\hat{H}_L = -\sum_{i=1}^N \hat{\sigma}^i_-\, \boldsymbol{d} \cdot \hat{\boldsymbol{E}}(\boldsymbol{r}_i) + \hat{\sigma}^i_+ \boldsymbol{d}^* \cdot \hat{\boldsymbol{E}}(\boldsymbol{r}_i)\,$, [c.f. Eq. (3) of the main text].

Here, $\boldsymbol{d}$ is the atomic dipole vector and the electric field operator is given by

$$\hat{\boldsymbol{E}}(\boldsymbol{r}_i) = i \sum_{i=1}^{N} \sum_{\boldsymbol{k},\lambda} \sqrt{\frac{2\omega_k}{V}} \boldsymbol{e}_\lambda(\boldsymbol{k}) \left(\hat{b}_\lambda(\boldsymbol{k}) e^{i\boldsymbol{k}\cdot\boldsymbol{r}_i} - \hat{b}^\dagger_\lambda(\boldsymbol{k}) e^{-i\boldsymbol{k}\cdot\boldsymbol{r}_i} \right), \tag{3}$$

where $\boldsymbol{e}_\lambda(\boldsymbol{k})$ and $\hat{b}^{(\dagger)}_\lambda(\boldsymbol{k})$ are the polarisation vector of the field and its annihilation (creation) operator, respectively. The system dynamics is then generically determined by the following master equation[26]

$$\frac{d}{dt}\rho_S(t) = -i[\hat{H}_S + \hat{H}_I, \rho_S(t)] + \sum_\omega \sum_{i,j} \left[\Gamma_{i,j}(\omega)(\hat{A}_j \rho_S(t) \hat{A}^\dagger_i - \hat{A}^\dagger_i \hat{A}_j \rho_S(t)) + \text{h.c.} \right], \tag{4}$$

where h.c. denotes the Hermitian conjugate. The $\hat{A}_i$ are the Lindblad operators given by $\hat{\sigma}^i_-$ and $\hat{\sigma}^i_+$, and $\Gamma_{i,j} = \int_0^\infty ds\, e^{i\omega s} \langle \boldsymbol{d}^* \cdot \hat{\boldsymbol{E}}(\boldsymbol{r}_i, s) \boldsymbol{d} \cdot \hat{\boldsymbol{E}}(\boldsymbol{r}_j, 0) \rangle$ is the spectral correlation tensor, which will be calculated in the following. We start by considering the expression

$$\langle \boldsymbol{d}^* \cdot \hat{\boldsymbol{E}}(\boldsymbol{r}_i, s) \boldsymbol{d} \cdot \hat{\boldsymbol{E}}(\boldsymbol{r}_j, 0) \rangle = Tr_{\mathcal{E}}[\boldsymbol{d}^* \cdot \hat{\boldsymbol{E}}(\boldsymbol{r}_i, s) \boldsymbol{d} \cdot \hat{\boldsymbol{E}}(\boldsymbol{r}_j, 0) \rho_{\mathcal{E}}] , \tag{5}$$

where $\rho_{\mathcal{E}}$ is taken as the thermal state of the environment, though allowing filtered thermal light later will not change the form of the result[27,28]. Generally, for a thermalised environment it is well known that[26]

$$\langle \hat{b}_\lambda(\boldsymbol{k}) \hat{b}_{\lambda'}(\boldsymbol{k'}) \rangle = \langle (\hat{b}^\dagger_\lambda(\boldsymbol{k}) \hat{b}^\dagger_{\lambda'}(\boldsymbol{k'}) \rangle = 0 , \tag{6}$$

$$\langle \hat{b}_\lambda(\boldsymbol{k}) \hat{b}^\dagger_{\lambda'}(\boldsymbol{k'}) \rangle = \delta_{\boldsymbol{k}\boldsymbol{k'}} \delta_{\hat{\lambda}\hat{\lambda}'} = (1 + n(\omega_k)) , \tag{7}$$

$$\langle \hat{b}^\dagger_\lambda(\boldsymbol{k}) \hat{b}_{\lambda'}(\boldsymbol{k'}) \rangle = \delta_{\boldsymbol{k}\boldsymbol{k'}} \delta_{\hat{\lambda}\hat{\lambda}'} = n(\omega_k) . \tag{8}$$

Using these, the spectral correlation tensor can written as:

$$\Gamma_{i,j} = \frac{2\pi}{V} \sum_{\boldsymbol{k},\lambda} (\boldsymbol{d} \cdot \boldsymbol{e}_\lambda(\boldsymbol{k}))^2 \omega_k \left((1 + n(\omega_k)) e^{i\boldsymbol{k}\cdot\boldsymbol{r}_{ij}} \int_0^\infty ds\, e^{-i(\omega_k - \omega)s} + e^{-i\boldsymbol{k}\cdot\boldsymbol{r}_{ij}} n(\omega_k) \int_0^\infty ds\, e^{i(\omega_k + \omega)s} \right) , \tag{9}$$

where $\boldsymbol{r}_{i,j}$ is the vector connecting atoms i and j. Converting the sum over $\boldsymbol{k}$ to an integral ($\omega_k = c|\boldsymbol{k}|$) yields

$$\frac{1}{V} \sum_{\boldsymbol{k}} \to \frac{1}{(2\pi)^3 c^3} \int_0^\infty d\omega_k\, \kappa(\omega_k) \omega_k^2 \int d\Omega\, , \tag{10}$$

where $\kappa(\omega)$ is the spectral density given by the density of states weighted by the coupling strength, $\kappa(\omega) = \sum_k |g_k|^2 \delta(\omega - \omega_k) \equiv \chi(\omega)|g(\omega)|^2$. The angular part of the integration gives a diffraction-type function:

$$F(\omega\, \boldsymbol{r}_{ij}) = \frac{8\pi}{3} \left(j_0(\omega\, |\boldsymbol{r}_{ij}|) + \frac{1}{2} \left(3\cos^2(\theta_{\boldsymbol{d}\,\boldsymbol{r}_{ij}}) - 1 \right) j_2(\omega\, |\boldsymbol{r}_{ij}|) \right) , \tag{11}$$

where $j_n(x)$ is the nth spherical Bessel function and the angle $\theta_{\boldsymbol{d}\,\boldsymbol{r}_{ij}}$ between the atomic dipoles and pairwise connection vectors is

$$\cos^2 \theta_{\boldsymbol{d}\,\boldsymbol{r}_{ij}} = \frac{|\boldsymbol{d} \cdot \boldsymbol{r}_{ij}^2|}{|\boldsymbol{d}^2||\boldsymbol{r}_{ij}{}^2|} . \tag{12}$$

Considering the geometry in Fig. 1 of the main text, we assume that all dipoles are parallel, and perpendicular to the plane defined by the ring. In this case, and for only nearest neighbour interactions, $\theta_{\boldsymbol{d}\,\boldsymbol{r}_{ij}}$ is independent of i and j. Thus Eq. (9) becomes

$$\Gamma_{i,j} = \frac{|d|^2}{(2\pi)^2 c^3} \int_0^\infty d\omega_k\, \kappa(\omega_k) \omega_k^3\, F(\omega_k r_{ij}) \left((1 + n(\omega_k)) \int_0^\infty ds e^{-i(\omega_k - \omega)s} + n(\omega_k) \int_0^\infty ds e^{i(\omega_k + \omega)s} \right) , \tag{13}$$

which we separate into its real and imaginary parts $\Gamma_{i,j} = \frac{1}{2}\gamma_{i,j}(\omega) + iS(\omega)$ with the help of the identity

$$\int_0^\infty ds\, e^{\pm i\epsilon s} = \pi\delta(\epsilon) \pm iP\frac{1}{\epsilon}. \tag{14}$$

The real terms $\gamma_{i,j}(\omega)$ derive from the δ-functions and give rise to the dissipative dynamics, i.e. optical transitions in this case. In the remaining term, $F(\omega_k\, r_{ij})$ is evaluated at $\omega_k = \pm\omega$. We are working in the small sample limit, where the wavelength of light is far longer than the size of our nanostructure ($\omega\, r_{ij} \approx 0$), and so $F(\omega\, r_{ij}) \approx 8\pi/3$. Hence $\gamma_{i,j}$ is independent of the atomic indices to a good approximation:

$$\gamma_{i,j}(\omega) \approx \gamma(\omega) = \frac{4\omega^3 |d|^2}{3c^3}\kappa(\omega)(1+n(\omega))\,. \tag{15}$$

The Planck distribution has the property that $n(-\omega) = -(1+n(\omega))$. Thus we can combine the terms arising from $\delta(\omega_k \pm \omega)$ and only run the sum over positive values. The second term on the righthand side of Eq. (4) thus becomes

$$\sum_{\omega>0}\sum_{i,j} \frac{4\omega^3|d|^2}{3c^3}\kappa(\omega)\left((1+n(\omega))(\hat{\sigma}_-^j\rho\hat{\sigma}_+^i - \frac{1}{2}\left\{\hat{\sigma}_+^i\hat{\sigma}_-^j,\rho\right\}) + n(\omega)(\hat{\sigma}_+^j\rho\hat{\sigma}_-^i - \frac{1}{2}\left\{\hat{\sigma}_-^i\hat{\sigma}_+^j,\rho\right\})\right)\,. \tag{16}$$

By simply assuming all transitions have the same frequency splitting ($\omega = \omega_A$), a vacuum environment state $n(\omega) = 0$ and switching to the collective operators to express the sums $\hat{J}_- = \sum_i \hat{\sigma}_-^i$, we reproduce the ordinary superradiance master equation.

We now turn to the imaginary part $S(\omega)$ of the spectral correlation tensor, which will be responsible for providing the detuning between different transitions; this is given by:

$$S(\omega) = \frac{|d|^2}{(2\pi)^2c^3} P\int_0^\infty d\omega_k\, \kappa(\omega_k)\omega_k^3\, F(\omega_k\, r_{ij})\left(\frac{1+n(\omega_k)}{\omega-\omega_k} + \frac{n(\omega_k)}{\omega_k+\omega}\right)\,. \tag{17}$$

The $i = j$ terms, for which $F(0) = 8\pi/3$, correspond to the ordinary Lamb shift of individual atom transitions; these can be accounted for by a renormalisation of the bare atomic frequency ω_A. By contrast, the $i \neq j$ terms correspond to the dipole-dipole interaction induced by the EM field. Evaluating this integral requires us to choose a specific form for the spectral density $\kappa(\omega)$. Here we consider two cases: first a flat spectral density, and second one that features a 'stop band' in the spectrum, blocking the 'bad' transition at frequency ω_{bad}. We begin with the former case. We first separate out the term that is independent of $n(\omega)$, the Lamb shift $S_L(\omega)$, and evaluate it. In the small sample limit ($\omega r_{ij} \ll 1$) we find

$$S_L(\omega) = \frac{d^2}{4\pi\epsilon_0 r_{ij}^3}\left[1 - 3\cos^2(\theta_{dr})\right]\,. \tag{18}$$

After separating out the Lamb shift, we are left with the divergent integral corresponding to the Stark shift:

$$S_s(\omega) = \frac{|d|^2}{(2\pi)^2c^3} P\int_0^\infty d\omega_k\, \kappa(\omega_k)\omega_k^3\, F(\omega_k r_{ij})\left(\frac{n(\omega_k)}{\omega-\omega_k} + \frac{n(\omega_k)}{\omega+\omega_k}\right)\,. \tag{19}$$

This is seldom evaluated in the literature and is usually assumed negligible. In this work we are primarily concerned with controlling $n(\omega)$ so that it is only significant for one mode, which has frequency ω_g. In this case we set $n(\omega_k) = \delta(\omega_k - \omega_g)$ and take the small sample limit:

$$S_s(\omega) = \lim_{r\omega_g \to 0} \frac{4\pi\omega\left(\sin(r\omega_g)\left(\cos(2\theta_{dr})\left(3 - r^2\omega_g^2\right) + r^2\omega_g^2 + 1\right) - r\omega_g(3\cos(2\theta_{dr})+1)\cos(r\omega_g)\right)}{r^3(\omega-\omega_g)(\omega+\omega_L)} = 0\,. \tag{20}$$

Hence we can neglect the Stark shift and only retain the Lamb shift. Returning to the other spectral density we consider in the main text, that with the stop band, we can express the stop band with the following, simplistic spectral density:

$$\kappa(\omega) = 1 - T(\omega_b, \sigma)\,, \tag{21}$$

where $T(\omega_b, \sigma)$ is the 'top hat' function centred on ω_b with a width σ. The factor of one produces the same result as for the flat spectral density $S_s(\omega)$. The top hat handles the effect of the gap $S_{gap}(\omega)$:

$$S(\omega) = S_s(\omega) - S_{gap}(\omega)\,. \tag{22}$$

The top hat has the effect of confining the integral to a window around ω_b:

$$S_{gap}(\omega) = \frac{|d|^2}{(2\pi)^2c^3} P\int_{\omega_b-\sigma}^{\omega_b+\sigma} d\omega_k\, \omega_k^3\, F(\omega_k r_{ij})\left(\frac{1+n(\omega_k)}{\omega-\omega_k} + \frac{n(\omega_k)}{\omega_k+\omega}\right)\,. \tag{23}$$

This can be evaluated to yield a lengthly, but straightforward expression. For an ideal gap ($\sigma \to 0$), $S_{gap}(\omega) = 0$ and hence can be neglected. Collating these results, we are left with a familiar expression for the strength of the interaction between two dipoles (18) multiplied by a hopping term introduced via the EM field:

$$\hat{H}_I = S_L(\omega) \sum_{i \neq j}^{N} \left(\hat{\sigma}_+^i \hat{\sigma}_-^j + \hat{\sigma}_-^i \hat{\sigma}_+^j \right) . \tag{24}$$

In the main text we consider the nearest neighbour limit of this expression, although this is not a necessity (see Supplementary Method 3). We know that the EM field operators interact with the system collectively, causing them to explore the ladder of Dicke states defined by:

$$|J, M\rangle = \sqrt{\frac{(J+M)!}{N!(J-M)!}} \hat{J}_-^{(J-M)} |ee....e\rangle . \tag{25}$$

The highly symmetric geometry of our system means that the hopping interaction (24) does not cause mixing of Dicke levels, but only shifts their energies (see main text). Thus an effective Hamiltonian for the subspace consisting of only the fully symmetric states of the Dicke ladder can be written as :

$$\hat{H}_S + \hat{H}_I = \frac{E_M}{2} \sum_{M=-J}^{J} |J, M\rangle\langle J, M| , \tag{26}$$

where E_M is the energy of the $|J, M\rangle$ state now including the shift defined in Eq. (10) of the main text. Instead of having a generic ladder operator $\hat{J}_\pm$ that moves any state $|J, M\rangle \to |J, M \pm 1\rangle$, with emission or absorption at ω_A, we now have to break up this operator, because the Dicke transitions are no longer degenerate in energy. The generic ladder operators are thus replaced by a sum over operators, which take us between specific Dicke states, sampling the spectral density at the requisite frequency ω_β:

$$\hat{L}_M = |J, M-1\rangle\langle J, M| , \tag{27}$$

which yields the result in the main text Eq. (7):

$$\dot{\rho} = -i[\hat{H}_S + \hat{H}_I, \rho] - \gamma \sum_\beta \kappa(\omega_\beta) \Big((n(\omega_\beta) + 1) D[\hat{L}_\beta]\rho + n(\omega_\beta) D[\hat{L}_\beta^\dagger]\rho \Big) . \tag{28}$$

VI. SUPPLEMENTARY METHOD 2: RESOLUTION OF FREQUENCY SHIFTS

The enhanced absorption and emission rate in the middle of the Dicke ladder implies an increased lifetime broadening. One might thus worry whether the detunings obtained courtesy of the hopping interaction are then still sufficient to completely resolve adjacent transitions. A simple analysis shows that this is indeed the case[25], and the natural width ($N^2\gamma$ around $M = 0$) remains smaller than the shift δ_ω provided the wavelength of the light is much greater than the size of the system. To see this, we shall assume that the transition is indeed well resolved, $N^2\gamma < \delta_\omega$, and show that is essentially equivalent to the 'small sample' condition, $r \ll \lambda$, which underlies the phenomenon of superradiance in the first place.

Using the definition of γ from as the free atom decay rate (see main text), the greatest broadening and smallest energy shift at $M = 0$ are, respectively,

$$N^2\gamma = \frac{8N^2\pi^2 d^2}{3\epsilon_0 \hbar \lambda^3} , \tag{29}$$

$$\delta_\omega = 4\frac{\Omega}{N-1} \approx \frac{d^2}{4\pi\hbar\epsilon_0 r^3} . \tag{30}$$

Substituting Eqs. (29) and (30), the inequality $N^2\gamma < \delta_\omega$ becomes

$$\frac{2N^3\pi^2 d^2}{3\epsilon_0\hbar\lambda^3} < \frac{d^2}{4\pi\hbar\epsilon_0 r^3} \quad \Longleftrightarrow \quad 2N\pi r < \lambda , \tag{31}$$

where the righthand side follows after cancellation of several variables followed by taking the qubic root. This is equivalent to $r \ll \lambda$, up to moderate numerical factor (when N is not too large), accounted for by relaxing '$\ll$' to '$<$'. For the present discussion, r is understood to be the nearest neighbour distance, having assumed energy shifts appropriate for only nearest neighbour interactions (for other interaction models, the detunings would be larger). We note that distinct shifted lines have also already been observed — and resolved — experimentally[29].

VII. SUPPLEMENTARY METHOD 3: INTERACTIONS BEYOND THE NEAREST NEIGHBOUR LIMIT

In the main text we assumed only nearest neighbour interactions are significant. For a symmetric ring geometry relaxing this condition leads to the same qualitative behaviour, but results in slightly larger detunings between adjacent transitions in the Dicke ladder. First, let us consider the opposite limit to the nearest neighbour case and allow all pairwise interactions with equal strength:

$$\langle J,M|\hat{H}_H\,|J,M\rangle = \langle J,M|\Omega\sum_{i\neq j}(\hat{\sigma}_+^i\hat{\sigma}_-^j + \hat{\sigma}_-^i\hat{\sigma}_+^j)\,|J,M\rangle\ , \tag{32}$$

$$\delta E_m = \Omega\langle J,M|\sum_{i\neq j}(\hat{\sigma}_+^i\hat{\sigma}_-^j + \hat{\sigma}_-^i\hat{\sigma}_+^j)\,|J,M\rangle\ , \tag{33}$$

which can be rewritten using the collective operators as follows

$$\delta E_m = \Omega\langle J,M|\hat{J}_+\hat{J}_- + \hat{J}_-\hat{J}_+ - \sum_i(\hat{\sigma}_+^i\hat{\sigma}_-^i + \hat{\sigma}_-^i\hat{\sigma}_+^i)\,|J,M\rangle\ . \tag{34}$$

The final two terms are added to remove the $i = j$ terms implicit in the $\hat{J}_+\hat{J}_-$ terms, which count the number of excited and unexcited atoms, respectively. Hence,

$$\delta E_m = \Omega(\langle J,M|\hat{J}_+\hat{J}_- + \hat{J}_-\hat{J}_+\,|J,M\rangle - 2J)\,. \tag{35}$$

The remaining two terms are easily calculated from Eq. (4) of the main text, yielding:

$$\delta E_m = 2\Omega(J^2 - M^2)\,. \tag{36}$$

Thus the energy shifts are the same as in the nearest neighbour case Eq. (10) but lack the factor$(J-1/2)^{-1}$. Therefore, unlike in the nearest neighbour limit, increasing the number of atoms does not reduce the size of the frequency shift, which could help in blocking the transition at $\omega_{\rm bad}$ and ensuring frequency selectivity of a trapping mechanism.

The actual ring geometry with all pairwise dipole interactions included will fall somewhere in between these two limits, depending on ring size. The operators involved in the interaction remain the same, but their weights are altered as the size of the ring changes. The symmetry of the ring dictates that each atom will be subject to the same set of interactions with the rest of the ring. The condition of interchangeability of atoms is thus met regardless of the specific interaction model (i.e. nearest neighbour, next nearest neighbour etc.). For all cases, the hopping interaction only causes shifts of a variable size between the two limits we have discussed above; the size of the shifts given a particular ring size and interaction model is readily obtained numerically.

VIII. SUPPLEMENTARY METHOD 4: THE IMPLICIT INTERACTION APPROACH

In the main text, the hopping interaction emerged from the derivation of the master equation, as a direct consequence of embedding the absorbers into a common electromagnetic environment. Alternatively, we could add an interaction to the Hamiltonian, which may either be mediated by virtual photon exchange or have some other physical origin. The initial Hamiltonian then reads:

$$\hat{H}_S = \omega_A\sum_{m=1}^{N}\hat{\sigma}_+^m\hat{\sigma}_-^m + \Omega\sum_{i,j}^{N}(\hat{\sigma}_+^i\hat{\sigma}_-^j + \hat{\sigma}_-^j\hat{\sigma}_+^i)\,. \tag{37}$$

Such a Hamiltonian is a diagonalised using the Jordan-Wigner transformation[30]. For example, a four atom system has eigenvalues:

$$\left\{0, \omega_A - 2\Omega, 2\omega_A - 2\sqrt{2}\Omega, 3\omega_A - 2\Omega, 4\omega_A\right\}\ , \tag{38}$$

resulting in the following transition frequencies:

$$\omega = \left\{\omega_A - 2\Omega, \omega_A - 2\Omega\left(1+\sqrt{2}\right), \omega_A + 2\Omega\left(-1+\sqrt{2}\right), \omega_A + 2\Omega\right\}\ . \tag{39}$$

These differ slightly from those derived using the approach in the main text. Crucially, however, the degeneracy of the transition frequencies is broken in a similar way as before, so that the discussion of environmental control for confining the dynamics to a specific transition remains valid. For small length scale linear systems it has been noted that superradiance dynamics are not significantly altered[30], when compared to the traditional field mediated interaction approach[25], used in the main text.

IX. SUPPLEMENTARY METHOD 5: NUMERICAL MODELS AND THE EFFECT OF DISORDER

In this Section we shall verify the result of the main article using numerical approaches. As a first step, we investigate the validity of the master equation framework described in the article. This is achieved with a Monte Carlo approach which allows the system to explore the entire Dicke ladder. In order to study disorder in the system Hamiltonian, we also present results from a completely independent model, which only builds on the physical Hamiltonian and makes no assumptions about the Dicke model being a good description of the system.

A. Monte Carlo

The Effective Two Level System (E2LS) model in the main text allowed us to reduce the complexity of the problem dramatically and made it analytically tractable. In order to verify this approach we here compare it to an independent numerical model: Supplementary Figure 3 shows excellent agreement between the E2LS model and Monte Carlo simulations of the master equation (28) [Eq. (7) of the main text] using QuTip[1]. Monte Carlo simulations of quantum systems use a time/memory tradeoff to allow large systems to be simulated. They do this by only storing and propagating the state vector rather than the entire density matrix, by averaging over many trajectories the results converge toward the dynamics that would have been obtained by numerical integrating the master equation as in the previous section. The numerical model uses a phenomenological trapping. The agreement with the E2LS can be made arbitrarily close with increasing numbers of trajectories. The same agreement was also seen for systems with larger numbers of atoms.

B. Numerical integration of Master Equation

We proceed by exploring the effect of disorder on both superradiance and superabsorption. To capture these effects we must use a more general, but less analytically informative approach to deriving the master equation than the one presented in the main text and derived in detail in Supplementary Method 1.

Starting with the implicit interaction model, we now allow the site energies to vary. We aim to simulate the effect of static energy disorder, which – to some extent – will be present in all physical implementations of superabsorption. To do this we draw the site frequencies ω_m from a Gaussian distribution with a mean given by the atomic frequency ω_A and a standard deviation given by σ. The system Hamiltonian is:

$$\hat{H}_S = \sum_{m=1}^{N} \omega_m \hat{\sigma}_+^m \hat{\sigma}_-^m + \Omega \sum_{i,j}^{N} (\hat{\sigma}_+^i \hat{\sigma}_-^j + \hat{\sigma}_-^j \hat{\sigma}_+^i) \,. \tag{40}$$

If the full trap model is to be used the extra terms (see Supplementary Method 6) are added. The Hamiltonian is then diagonalised numerically and the eigenoperators determined which project the dipole operator on the eigenspace of $\hat{H}_S$:

$$\hat{A}(\omega) = \sum_{e,e'} \delta(\omega = e - e') |e\rangle\langle e| (\hat{J}_- + \hat{J}_+) |e'\rangle\langle e'| \,, \tag{41}$$

where $|e\rangle\langle e|$ is a projector onto the a given eigenstate of the Hamiltonian (37). $\hat{A}_\alpha(\omega)$ is therefore an eigenoperator of $\hat{H}_S$, which causes a transition between two eigenstates of $\hat{H}_S$ sampling the environment at frequency ω. We construct the new master equation analogously to Eq. (2):

$$\frac{d}{dt}\tilde{\rho}_{\mathcal{S}}(t) = -i \int_0^\infty dt' \mathrm{Tr}_{\mathcal{E}} [\tilde{H}_I(t), [\tilde{H}_I(t-t'), \tilde{\rho}_{\mathcal{S}}(t) \otimes \tilde{\rho}_{\mathcal{E}}] \,, \tag{42}$$

and the standard procedure[26] straightforwardly yields

$$\frac{d}{dt}\rho_S(t) = \sum_{\omega,\omega'} \sum_{\alpha,\beta} e^{i(\omega-\omega')t} \Gamma_{\alpha,\beta}(\omega) (\hat{A}_\beta(\omega) \rho_S(t) \hat{A}_\alpha^\dagger(\omega') - \hat{A}_\alpha^\dagger(\omega') \hat{A}_\beta(\omega) \rho_S(t)) + \text{h.c.} \,. \tag{43}$$

Equation (43) could directly be solved numerically, but this is extremely computationally expensive even for small disordered systems, due the pathological scaling in the number of terms arising from the summation over ω, ω', α and β. To overcome this difficulty, (43) is traditionally simplified using the rotating wave approximation (RWA). This means neglecting the non-secular terms (where $\omega \neq \omega'$), leading to the canonical 'quantum optical master equation':

$$\frac{d}{dt}\rho_S(t) = \sum_{\omega}\sum_{\alpha,\beta}\Gamma_{\alpha,\beta}(\omega)(\hat{A}_\beta(\omega)\rho_S(t)\hat{A}^\dagger_\alpha(\omega) - \hat{A}^\dagger_\alpha(\omega)\hat{A}_\beta(\omega)\rho_S(t)) + \text{h.c.}\ . \tag{44}$$

In the case of ordinary superradiance, with no dipole-dipole interaction and consequent shifts, this step is unproblematic because there is in fact only one transition frequency (ω_A). However, for all other cases, care must be taken to apply the approximation selectively only where it is strictly justified. The RWA assumes that the fast oscillating terms $e^{i(\omega-\omega')t}$ effectively average to out to zero over the timescale relevant to the system dynamics τ_R. The fastest system dynamics timescale is given by the reciprocal of the lowest non-zero transition frequency ω_{min}^{-1}. However, there is some subtlety to applying the RWA to collective transitions in disordered systems: Neglecting all non-secular terms instantly imposes the independent exponential decay type behaviour on the system. Applying no restriction is extremely computationally expensive, given that disorder calculation must be repeated many times and averaged to produce meaningful results. Removing too many non-secular terms causes an overestimation of the debilitating effect of disorder on the system: the dynamics to abruptly change from collective to independent behaviour with even the smallest amount of disorder. This is true of the commonly used order of magnitude separation between τ_R and ω_{min}^{-1}. Instead the criterion should made steadily more stringent until convergence is attained. By retaining these terms our model can smoothly interpolate between the limits of collective behaviour (superradiance/superabsorption) and independent exponential decay, which must emerge for strongly disordered systems. The resulting equation is then integrated numerically using the QuTip package[1]. The numerical calculation is then repeated many times in order to obtain the an ensemble average over different instances of disorder and smooth out the random oscillations introduced by any given instance.

Supplementary Fig. 4 shows the effect of disorder on superradiance. As expected both plots show a certain robustness to disorder. This is because the increased transition rates produced by superradiance serve to counterbalance the effect of disorder: the faster rate broadens the natural linewidth of the transitions, effectively removing the distinguishability of atoms introduced by the disorder. The model with dipole-dipole interactions shows greater robustness, as might be expected because its eigenstates are intrinsically delocalised, whereas with no interactions there is less of a barrier to the localisation introduced by σ. Supplementary Fig. 5 shows the effect of disorder on superabsorption. As expected from the argument above superabsorption also shows excellent robustness to disorder. It is slightly less robust than superradiance, because it relies on the Dicke ladder shifts not being too heavily altered by the level of static disorder characterised by σ. The ratio of static disorder to interaction strength needed for superabsorption, is well met by current experimental systems as detailed in Supplementary Table 1.

X. SUPPLEMENTARY METHOD 6: TRAPPING

In the main text we describe the trapping process using a Lindblad operator, which takes the system from the state $|J,0\rangle \rightarrow |J,-1\rangle$ by irreversibly removing one exciton from the system. Numerous microscopic mechanisms can be conceived of that would produce this effect; all will have to involve a collective coupling of the atoms of the ring, followed by a process (through coupling to a wider external environment) which prevents the return of exciton to the primary system, or at least renders it very unlikely.

Here, we give an account of the simplest scenario one can envision: all atoms of the ring are coupled to a 'trap' atom at the centre, whose role is to first localise the energy and then irreversibly remove it. This simple trapping model is schematically depicted in Supplementary Fig. 2: The trap atom possesses a transition frequency $\omega_{\text{trap}} \approx \omega_{\text{good}}$ and is coupled to the ring by a field mediated hopping interaction of strength g (i.e. the same type of interaction which couples the ring's atoms to each other). The Hamiltonian for such a set up is:

$$\hat{H}_{\text{T}} = g(\hat{J}_+\hat{\sigma}^T_- + \hat{J}_-\hat{\sigma}^T_+) + \omega_{\text{trap}}\hat{\sigma}^T_+\hat{\sigma}^T_-\,, \tag{45}$$

where the superscript T denotes the trap site, g is the strength of the coupling between the ring and the trap, and ω_{trap} is the trap's transition frequency, which ideally matches ω_{good}. An exciton 'hopping' onto the trap site is subjected to an irreversible decay with rate Γ_{trap}, e.g. by being linked to a chain of exciton sites acting as a wire or lead. In natural light harvesting systems the trap would be the reaction centre and the decay a photochemical process.

When the trapping rate Γ_{trap} is sufficiently fast only negligible population exists in the 2LS forming the trap, hence its effect can, to a good approximation, be considered that of a Lindblad operator acting on the main system. For a slower rate Γ_{trap} a decaying Rabi oscillation may take place, moving the exciton back and forth between trap and ring. However, the presence or absence of these oscillations does not significantly affect the superabsorption process.

This simplistic trapping model introduces an extra contribution to the rate of loss from the E2LS: the finite lifetime $1/\Gamma_{\text{trap}}$ means the energy of the trap is not perfectly sharply defined, reducing the trap's frequency selectivity. Thus, it will occasionally also accept energy from the 'bad' transition ω_{bad}, which increases the effective loss rate Γ_{loss}. However, one can optimise the parameters (Γ_{trap} , g, ω_{trap}) to minimise this undesirable side effect, whilst still meeting the condition that $\Gamma_{\text{trap}} > \Gamma_{\text{emit}}$. With the addition of the trapping terms (45) the master equation is numerically solved using the method described in Supplementary Method 5. We compare this against the analytical expression for the trapping rate that can be derived for the E2LS model described in the main text:

$$\rho_{\text{M}=0}(t) = \frac{\Gamma_{\text{absorb}}}{\sqrt{\Gamma_{\text{total}}^2 - 4\Gamma_{\text{loss}}(\Gamma_{\text{emit}} + \Gamma_{\text{trap}})}} \times \tag{46}$$

$$\left(e^{-\frac{1}{2}t\left(\Gamma_{\text{total}} - \sqrt{\Gamma_{\text{total}}^2 - 4\Gamma_L(\Gamma_{\text{emit}} + \Gamma_{\text{trap}})}\right)} - e^{-\frac{1}{2}t\left(\Gamma_{\text{total}} + \sqrt{\Gamma_{\text{total}}^2 - 4\Gamma_L(\Gamma_{\text{emit}} + \Gamma_{\text{trap}})}\right)}\right),$$

$$I_{\text{trap}}(t) = \Gamma_{\text{trap}}\, \rho_{\text{M}=0}(t), \tag{47}$$

where $\Gamma_{\text{total}} = \Gamma_{\text{absorb}} + \Gamma_{\text{emit}} + \Gamma_{\text{loss}} + \Gamma_{\text{trap}}$ and with Γ_{trap} referring to the effective rate of a trapping Lindblad operator, which is related, but generally not necessarily equal to the decay rate of the trap site in the full model. This is due to the effect additional parameters such as the coupling strength g. By accounting for this and the additional contribution to Γ_{loss} described above, we can compare the trap site model against the E2LS. Supplementary Fig. 1 shows the results of this comparison. The analytical results for the E2LS can thus provide an adequate and simple qualitative description of the dynamics that is brought about by a more complex and realistic trapping model.

XI. SUPPLEMENTARY REFERENCES

1. Johansson, J., Nation, P. & Nori, F. Qutip 2: A python framework for the dynamics of open quantum systems. *Comput. Phys. Commun.* **184**, 1234 – 1240 (2013).
2. Dolan, P. R., Hughes, G. M., Grazioso, F., Patton, B. R. & Smith, J. M. Femtoliter tunable optical cavity arrays. *Opt. Lett.* **35**, 3556–3558 (2010).
3. Akahane, Y., Asano, T., Song, B.-S. & Noda, S. High-q photonic nanocavity in a two-dimensional photonic crystal. *Nature* **425**, 944–947 (2003).
4. Tanabe, T., Notomi, M., Kuramochi, E., Shinya, A. & Taniyama, H. Trapping and delaying photons for one nanosecond in an ultrasmall high-q photonic-crystal nanocavity. *Nature Photon.* **1**, 49–52 (2007).
5. Englund, D. *et al.* Controlling the spontaneous emission rate of single quantum dots in a two-dimensional photonic crystal. *Phys. Rev. Lett.* **95**, 013904 (2005).
6. Kress, A. *et al.* Manipulation of the spontaneous emission dynamics of quantum dots in two-dimensional photonic crystals. *Phys. Rev. B.* **71**, 241304 (2005).
7. Vahala, K. J. Optical microcavities. *Nature* **424**, 839–846 (2003).
8. Wang, Q., Stobbe, S. & Lodahl, P. Mapping the local density of optical states of a photonic crystal with single quantum dots. *Phys. Rev. Lett.* **107**, 167404 (2011).
9. Noda, S., Fujita, M. & Asano, T. Spontaneous-emission control by photonic crystals and nanocavities. *Nature Photon.* **1**, 449–458 (2007).
10. Leistikow, M. *et al.* Inhibited spontaneous emission of quantum dots observed in a 3d photonic band gap. *Phys. Rev. Lett.* **107**, 193903 (2011).
11. Frölich, A., Fischer, J., Zebrowski, T., Busch, K. & Wegener, M. Titania woodpiles with complete three-dimensional photonic bandgaps in the visible. *Adv. Mater.* **25**, 3588–3592 (2013).
12. Dorfman, K. E., Voronine, D. V., Mukamel, S. & Scully, M. O. Photosynthetic reaction center as a quantum heat engine. *Proceedings of the National Academy of Sciences* **110**, 2746–2751 (2013). http://www.pnas.org/content/110/8/2746.full.pdf+html.
13. Creasey, M., Lee, J.-H., Wang, Z., Salamo, G. J. & Li, X. Self-assembled InGaAs quantum dot clusters with controlled spatial and spectral properties. *Nano Lett.* **12**, 5169–5174 (2012).
14. Unold, T., Mueller, K., Lienau, C., Elsaesser, T. & Wieck, A. D. Optical control of excitons in a pair of quantum dots coupled by the dipole-dipole interaction. *Phys. Rev. Lett.* **94**, 137404 (2005).

15. Lovett, B. W., Reina, J. H., Nazir, A. & Briggs, G. A. D. Optical schemes for quantum computation in quantum dot molecules. *Phys. Rev. B.* **68**, 205319 (2003).
16. O'Sullivan, M. C. *et al.* Vernier templating and synthesis of a 12-porphyrin nano-ring. *Nature* **469**, 72–75 (2011).
17. Kobayashi, T. *J-Aggregates.* No. v. 2 in J-aggregates (World Scientific, 2012).
18. Bassani, G. & Agranovich, V. *Electronic Excitations in Organic Based Nanostructures.* Thin Films and Nanostructures (Elsevier Science, 2003).
19. K., S. S., Alexander, E., Stéphanie, V. & Alán, A.-G. Photonics meets excitonics: natural and artificial molecular aggregates. *Nanophotonics* **2**, 21 (2013).
20. Purcell, E. M., Torrey, H. C. & Pound, R. V. Resonance absorption by nuclear magnetic moments in a solid. *Phys. Rev.* **69**, 37–38 (1946).
21. Lu, S. & Madhukar, A. Nonradiative resonant excitation transfer from nanocrystal quantum dots to adjacent quantum channels. *Nano Lett.* **7**, 3443–3451 (2007). http://pubs.acs.org/doi/pdf/10.1021/nl0719731.
22. Lu, S. *et al.* Photocurrent induced by nonradiative energy transfer from nanocrystal quantum dots to adjacent silicon nanowire conducting channels: Toward a new solar cell paradigm. *Nano Lett.* **9**, 4548–4552 (2009). PMID: 19856942, http://pubs.acs.org/doi/pdf/10.1021/nl903104k.
23. Agranovich, V., Gartstein, Y. N. & Litinskaya, M. Hybrid resonant organic–inorganic nanostructures for optoelectronic applications. *Chem. Rev.* **111**, 5179–5214 (2011).
24. Dorn, A., Strasfeld, D. B., Harris, D. K., Han, H.-S. & Bawendi, M. G. Using nanowires to extract excitons from a nanocrystal solid. *ACS Nano* **5**, 9028–9033 (2011). http://pubs.acs.org/doi/pdf/10.1021/nn203227t.
25. Gross, M. & Haroche, S. Superradiance: An essay on the theory of collective spontaneous emission. *Phys. Rep.* **93**, 301–396 (1982).
26. Breuer, H. P. & Petruccione, F. *The Theory of Open Quantum Systems* (Oxford University, 2002).
27. Pechen, A. Engineering arbitrary pure and mixed quantum states. *Phys. Rev. A.* **84**, 042106 (2011).
28. Accardi, L., Lu, Y. G. & Volovich, I. *Quantum theory and its stochastic limit* (Springer Verlag, 2002).
29. Wang, H., Zheng, X., Zhao, F., Gao, Z. & Yu, Z. Superradiance of high density Frenkel excitons at room temperature. *Phys. Rev. Lett.* **74**, 4079–4082 (1995).
30. Benedict, M. G. *Super-radiance: Multiatomic coherent emission* (Taylor & Francis, 1996).
